# Iron limitation of heterotrophic bacteria in the California Current System tracks relative availability of organic carbon and iron

**DOI:** 10.1093/ismejo/wrae061

**Published:** 2024-04-16

**Authors:** Lauren E Manck, Tyler H Coale, Brandon M Stephens, Kiefer O Forsch, Lihini I Aluwihare, Christopher L Dupont, Andrew E Allen, Katherine A Barbeau

**Affiliations:** Geosciences Research Division, Scripps Institution of Oceanography, University of California San Diego, La Jolla, CA 92093, United States; Flathead Lake Biological Station, University of Montana, Polson, MT 59860, United States; Integrative Oceanography Division, Scripps Institution of Oceanography, University of California San Diego, La Jolla, CA 92093, United States; Ocean Sciences Department, University of California Santa Cruz, Santa Cruz, CA 95064, United States; Department of Environment and Sustainability, J. Craig Venter Institute, La Jolla, CA 92037, United States; Geosciences Research Division, Scripps Institution of Oceanography, University of California San Diego, La Jolla, CA 92093, United States; Institute of Oceanography, National Taiwan University, Taipei, 106, Taiwan; Geosciences Research Division, Scripps Institution of Oceanography, University of California San Diego, La Jolla, CA 92093, United States; Geosciences Research Division, Scripps Institution of Oceanography, University of California San Diego, La Jolla, CA 92093, United States; Department of Environment and Sustainability, J. Craig Venter Institute, La Jolla, CA 92037, United States; Department of Human Health, J. Craig Venter Institute, La Jolla, CA 92037, United States; Department of Synthetic Biology, J. Craig Venter Institute, La Jolla, CA 92037, United States; Integrative Oceanography Division, Scripps Institution of Oceanography, University of California San Diego, La Jolla, CA 92093, United States; Department of Environment and Sustainability, J. Craig Venter Institute, La Jolla, CA 92037, United States; Geosciences Research Division, Scripps Institution of Oceanography, University of California San Diego, La Jolla, CA 92093, United States

**Keywords:** marine, heterotrophic bacteria, iron, carbon, transcriptomics, biogeochemistry, biological carbon pump, California Current System

## Abstract

Iron is an essential nutrient for all microorganisms of the marine environment. Iron limitation of primary production has been well documented across a significant portion of the global surface ocean, but much less is known regarding the potential for iron limitation of the marine heterotrophic microbial community. In this work, we characterize the transcriptomic response of the heterotrophic bacterial community to iron additions in the California Current System, an eastern boundary upwelling system, to detect *in situ* iron stress of heterotrophic bacteria. Changes in gene expression in response to iron availability by heterotrophic bacteria were detected under conditions of high productivity when carbon limitation was relieved but when iron availability remained low. The ratio of particulate organic carbon to dissolved iron emerged as a biogeochemical proxy for iron limitation of heterotrophic bacteria in this system. Iron stress was characterized by high expression levels of iron transport pathways and decreased expression of iron-containing enzymes involved in carbon metabolism, where a majority of the heterotrophic bacterial iron requirement resides. Expression of iron stress biomarkers, as identified in the iron-addition experiments, was also detected *in*  *situ*. These results suggest iron availability will impact the processing of organic matter by heterotrophic bacteria with potential consequences for the marine biological carbon pump.

## Introduction

Iron (Fe) is an essential cofactor in many enzymes facilitating fundamental life processes such as photosynthesis, respiration, and nitrogen fixation. Dissolved iron (dFe) is therefore a necessary micronutrient for all microbial growth in the marine environment and is tightly linked to the cycling of carbon and other macronutrients [1]. However, low solubility coupled with enhanced biological uptake of Fe in the surface ocean results in pico- to nanomolar concentrations of dFe across the global surface ocean and limits primary production by photoautotrophs in more than one-third of the surface ocean [2]. One such region, the California Current System (CCS), is an eastern boundary current where upwelled nutrients fuel high levels of primary production [3]. However, a low supply of Fe relative to nitrate (NO_3_^−^) during upwelling events can drive the phytoplankton community to Fe limitation [4]. This results in high nutrient-low chlorophyll-like regions where NO_3_^−^ accumulates in surface waters due to incomplete utilization by the Fe-limited phytoplankton community. More recently, Fe limitation within the southern sector of the CCS has been documented [5, 6], and experimental evidence suggests that Fe limitation at the deep chlorophyll maximum (DCM) is a persistent and widespread feature of this system [7].

Although the effects of nutrient limitation on primary production in the CCS have been well documented, much less is known about the factors controlling heterotrophic bacterial activity, including the potential for Fe limitation. Marine heterotrophic bacteria also have significant Fe requirements, possibly exceeding those of marine phytoplankton [8–15]. Most of this Fe resides within proteins driving central carbon metabolism, such as those of glycolysis, the citric acid cycle, and the respiratory electron transport chain, where it facilitates essential redox reactions [16]. This indicates an important link between Fe availability to heterotrophic bacteria and the efficiency of their carbon metabolism. It is now recognized that heterotrophic bacteria are a key determinant in the fate of fixed carbon within the marine environment [17], acting as a major control on the attenuation of particulate organic carbon (POC) produced in the surface ocean [18–20]. Despite the small spatial coverage of eastern boundary current systems such as the CCS, their disproportionate levels of primary production make them significant contributors to global biogeochemical cycling and marine food webs [21]. Understanding controls on the activity of heterotrophic bacteria will be an important step in characterizing the efficiency of the biological carbon pump in these systems, the transfer of energy to higher trophic levels, and the net effects of eastern boundary currents on global biogeochemical cycles. Given the significant role that Fe plays in carbon metabolism and its limited availability in the marine environment, characterizing “both” the Fe and carbon requirements of heterotrophic bacteria will be critical to this understanding.

Fe limitation of heterotrophic marine bacteria has been assessed in a number of studies, both in the field [15, 22–27] and with cultured isolates [8, 9, 13, 28–30]. Laboratory studies have shown that Fe-limited bacterial strains generally exhibit decreased rates of respiration, growth, and Fe:C ratios compared to Fe-replete cultures [8, 9, 13, 29]. Field studies have shown varied responses of this community to Fe additions [27]. However, assessing Fe limitation of the heterotrophic community *in*  *situ* can be challenging given that Fe limitation of primary producers can indirectly affect the nutritional status and growth response of the heterotrophic microbial population. A reduced supply of fixed carbon due to photoautotrophic Fe limitation may result in a heterotrophic bacterial community that is carbon-limited or co-limited by carbon and Fe. Therefore, studies need to be designed to distinguish between carbon and Fe limitation of the heterotrophic bacterial community *in*  *situ*. Previous studies relying on bulk growth indicators have attempted to isolate the heterotrophic bacterial response to Fe availability by conducting growth experiments in the dark to eliminate photosynthetic activity or by removing the confounding effects of carbon limitation with the addition of labile organic substrates. However, by doing so, these experiments are no longer reflective of *in*  *situ* environmental conditions or interactions among the microbial community, making broader conclusions difficult to achieve.

**Figure 1:**
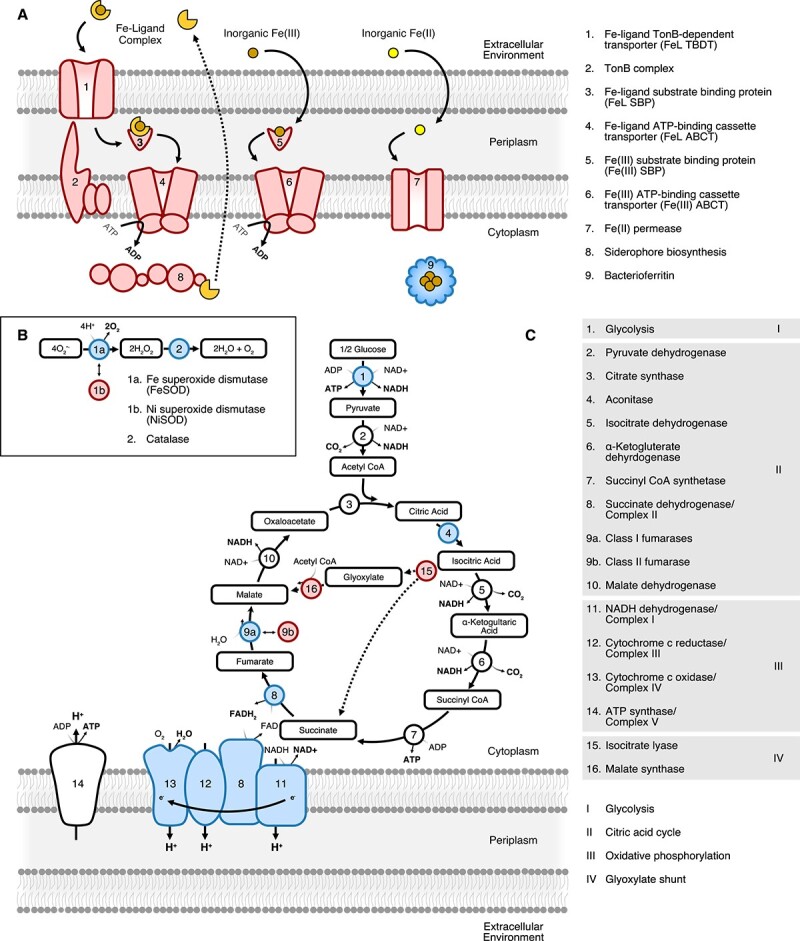
Summary of Fe stress biomarkers in heterotrophic marine bacteria identified in culture studies. (A) Simplified view of components of the three main Fe transport systems found within marine heterotrophic bacteria for the acquisition of organically complexed Fe (FeL), inorganic Fe(III), and inorganic Fe(II). Although only a generic transport system for the acquisition of FeL is displayed, a specific TBDT, SBP, and ABCT are necessary for each distinct FeL complex, which means that a high diversity of specific FeL transporters may be present within a bacterial community. FeL complexes include exogenously produced ligands as well as siderophores, strong Fe-binding ligands produced specifically by heterotrophic bacteria for Fe acquisition. The Fe-storage protein, bacterioferritin, enables luxury uptake of Fe and can significantly contribute to cellular Fe quotas in heterotrophic bacteria. (B) Pathway for management of cellular oxidative stress utilizing the Fe-containing enzymes superoxide dismutase and catalase, highlighted in blue. A nickel-containing superoxide dismutase is a non-Fe containing metabolic replacement present in some species. (C) Simplified summary of central carbon metabolism within a cell represented by glycolysis, the citric acid cycle, and oxidative phosphorylation with Fe-containing enzymes highlighted in blue. The individual steps of glycolysis are not displayed and specific pathways vary across species but can include Fe-containing enzymes such as ﻿6-phosphogluconate dehydratase in the ﻿Entner-Doudoroff pathway. The Fe-containing succinate dehydrogenase complex is a component of both the citric acid cycle and oxidative phosphorylation. Class II fumarases (fumarase c) are Fe-free enzymes which can serve as metabolic replacements for class I fumarases (fumarase a and b). The glyoxylate shunt, an alternative to the traditional citric acid cycle which bypasses the loss of carbon as CO_2_, is marked with dashed arrows. Flavodoxin, an Fe-free metabolic replacement for ferredoxin within photosynthetic electron transfer reactions, is discussed in the text but is not pictured here as it is specific to *Cyanobacteria*. Across all panels, Fe-containing proteins are highlighted in blue, and the expression of genes encoding these proteins has been observed to be decreased in marine bacteria under Fe-limiting conditions in culture studies. In contrast, proteins that have been observed to have increased expression under low-Fe conditions in culture studies are highlighted in red and include the Fe transport systems, non-Fe containing metabolic replacements, and enzymes of the glyoxylate shunt.

High-throughput sequencing of transcriptomes can query Fe limitation in the marine heterotrophic bacterial community independently of the photoautotrophic community, thus providing insight on the *in*  *situ* nutritional status of heterotrophic bacteria. Like other microorganisms, heterotrophic bacteria have molecular strategies for coping with limited Fe availability and the wide array of chemical forms in which it can be found in the marine environment [31, 32]. Culture studies have identified genetic biomarkers of these molecular strategies, which are differentially expressed by heterotrophic bacteria in consistent and unique patterns under low-Fe conditions (Fig. 1). These biomarkers broadly fall into three categories—Fe acquisition pathways, Fe-containing enzymes, and Fe-free metabolic replacements. The majority of dFe in the ocean is complexed by a pool of highly diverse organic ligands [33] (generally referred to as FeL), but trace amounts of inorganic Fe(III) are also present [34], and inorganic Fe(II) can accumulate in low-oxygen environments [35]. Fe speciation, therefore, acts as a strong control on Fe bioavailability, and heterotrophic bacteria must utilize a specific cellular transport system to access each of these forms of Fe (Fig. 1) [32, 36, 37]. In culture, these transport systems are consistently observed to be highly expressed under Fe-limiting conditions [13, 30, 38–41]. Once acquired by a bacterial cell, Fe is primarily found as a cofactor in enzymes of central carbon metabolism as well as within pathways for managing oxidative stress (Fig. 1). In culture, Fe-limiting conditions result in reduced expression of enzymes with Fe-containing cofactors as well as increased expression of Fe-free metabolic replacements [13, 30]. Combined, the expression patterns of such biomarkers in response to Fe availability can detect Fe stress in the heterotrophic bacterial community independently from that of the photosynthetic community and allow us to distinguish between multiple types of nutrient limitation [42], increasing our understanding of the nutritional status of the heterotrophic bacterial community *in*  *situ*.

## Materials and methods

### Study site

**Figure 2:**
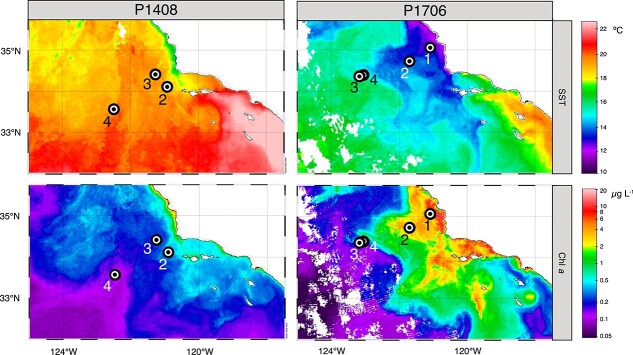
CCE LTER study region during the P1408 and P1706 process cruises. Plots display monthly averages of satellite derived sea surface temperature (SST, °C) and chlorophyll *a* concentrations (Chl *a*, μg L^−1^) during August 2014 (P1408) and June 2017 (P1706). Stations where on-array Fe-addition experiments were conducted are marked with a black circle and labeled according to the corresponding Cycle number.

The current study was conducted in the southern portion of the CCS (Fig. 2). Samples were collected during two California Current Ecosystem Long Term Ecological Research (CCE LTER) process cruises, P1408 and P1706, taking place from 08 August – 09 September 2014 aboard the R/V *Melville* and aboard the R/V *Roger Revelle* between 03 June – 30 June 2017. Sampling was conducted in a Lagrangian fashion within a single water mass over the course of 2 to 4 days [43]. Each sampling period within a single water mass has been termed a Cycle, as referred to throughout the text. During each Cycle, the water mass was tracked by the deployment of a drifter array with a subsurface drogue centered at 15 m. Samples were collected from Cycles 2 through 4 during P1408 which individually sampled three distinct regions and productivity regimes. During P1706, samples were collected from Cycles 1 through 4 which collectively captured a recently upwelled water mass from its origin at the coast as it aged and moved offshore. See the Supplemental Materials and Methods for detailed descriptions of sampling procedures and analyses of biogeochemical parameters.

### On-array Fe-addition incubations

On both the P1408 and P1706 cruises, Fe-addition experiments were conducted *in*  *situ* on the drifter array to assess the response of the microbial community to Fe additions via changes in gene expression. Whole seawater was collected from the DCM (P1408) or surface mixed-layer (P1706) using a powder-coated rosette equipped with Niskin-X bottles (Ocean Test Equipment) deployed on a coated hydrowire and subsequently processed inside a Class 100 clean van. These depths were targeted on respective cruises using residual *in*  *situ* NO_3_^−^ concentrations as an indicator of potential Fe limitation within the phytoplankton community. Upon collection of seawater, replicate *in*  *situ* samples were immediately processed by filtering ~2.7 L of whole seawater onto 0.2 μm Sterivex filters (MilliporeSigma), which were stored in liquid nitrogen until processing onshore. At the same time, incubation experiments were initiated by dispensing whole seawater into 1 L acid-cleaned (trace metal grade hydrochloric acid) polycarbonate bottles (Nalgene). A final concentration of 10 nmol L^−1^ FeCl_3_ was added to replicate treatment bottles representing typical dFe concentrations of upwelled water masses within the CCS [44]. The remaining replicate bottles were left as unamended controls. Treatment bottles and control bottles were each prepared in duplicate on P1408 and in triplicate on P1706. Bottles were sealed and secured in mesh bags to the drifter array at the depth at which water was initially collected (Table 1) and incubated at *in*  *situ* temperatures and light levels for 24 hours. Upon retrieval of the bottles, samples were immediately processed for RNA preservation by filtering the entire 1 L of seawater onto 0.2 μm Sterivex filters, which were stored in liquid nitrogen until processing onshore.

**Table 1:** *In*  *situ* environmental conditions at the onset of each Fe-addition experiment conducted during the P1408 (deep chlorophyll maximum) and P1706 (surface mixed-layer) CCE LTER process cruises.

**Cruise**	**Cycle**	**Latitude** **(°N)**	**Longitude** **(°W)**	**Depth** **(m)**	**POC** **(μmol L** ^**−1**^ **)**	**Chl *a* (μg L** ^**−1**^ **)**	**BCP** **(μmol L** ^**−1**^ **day**^**−1**^**)**	**dFe** **(nmol L** ^**−1**^ **)**	**NO**_**3**_^**−**^ **(μmol L** ^**−1**^ **)**	**Si** **(μmol L** ^**−1**^ **)**	**NO**_**3**_^**−**^: **dFe** **(μmol:nmol)**	**Si:N** **(mol:mol)**	**POC:dFe** **(μmol:nmol)**
**P1408**	**2**	34.11	120.92	35	6.71	1.10	0.07	0.33	1.97	1.70	5.89	0.86	20.08
	**3**	34.41	121.27	35	7.06	0.87	0.06	0.28	4.43	3.44	15.67	0.78	24.97
	**4**	33.57	122.49	60	3.07	0.33	0.06	0.23	0.13	2.39	0.56	18.38	13.31
**P1706**	**1**	35.07	121.09	12	18.13	4.65	0.51	2.11	12.09	12.03	5.72	0.99	8.58
	**2**	34.73	121.71	12	41.57	3.48	0.42	0.15	7.95	3.59	51.94	0.45	271.72
	**3**	34.36	123.18	12	17.31	1.37	0.19	0.37	3.95	3.15	10.59	0.80	46.35
	**4**	34.40	123.07	30	9.73	0.65	0.27	0.26	5.55	2.75	21.31	0.50	37.35

### RNA library preparation, sequencing, and bioinformatic analysis

RNA was extracted using a NucleoMag RNA kit (Macherey Nagel), with the lysis step performed inside the Sterivex unit. Lysate was transferred to a 96-well plate and the remainder of the protocol was performed on an epMotion liquid handling workstation (Eppendorf). RNA was analyzed on a TapeStation system (Agilent) using the high-sensitivity RNA ScreenTape assay. Ribosomal RNA was removed using RiboZero Magnetic kits (Epicenter) following the manufacturer’s low input protocol. cDNA was synthesized using the Ovation RNA-seq System V2 (NuGNE), and Agencourt RNAClean XP beads were used for cDNA purification. cDNA was fragmented using the Covaris E210 focused ultrasonicator, targeting 300 bp fragments. Library preparation was conducted with the Ovation Ultralow System V2 (NuGEN). After end repair, ligation, and amplification, libraries were quantified by qPCR with the KAPA Library Quantification Kit on the 7900HT Fast Real-time PCR System (Applied Biosystems). Pooled libraries were sequenced on a HiSeq 4000 platform (Illumina) using a 2×150 bp paired-end sequencing protocol at the Institute for Genomic Medicine at the University of California, San Diego.

Metatranscriptomes were constructed using the RNAseq Annotation Pipeline v0.4 (RAP) as described previously [45], and *ab initio* open-reading frames (ORFs) were predicted. Individual libraries were then merged to create a single assembly of the entire dataset used for downstream taxonomic and functional annotations. ORFs were annotated via BLASTP [46] to the phyloDB protein database, and pfam and Kyoto Encyclopedia of Genes and Genomes (KEGG) annotations were used for functional identifications. Taxonomy was assigned to ORFs based on a lineage probability index [47]. The terms ORF and gene will be used interchangeably throughout the text. Differential expression under varying experimental conditions was assessed using DESeq2 [48] within the R environment [49] and was considered significant for fold-change values with an *FDR* < 0.05 (Benjamini–Hochberg adjusted *P* value). ORF abundances from *in*  *situ* samples were normalized using the variance stabilizing transformation (vst) function in DESeq2 before downstream ordination and clustering analyses using the vegan package [50] within R.

## Results

### *In*  *situ* biogeochemical conditions

**Figure 3:**
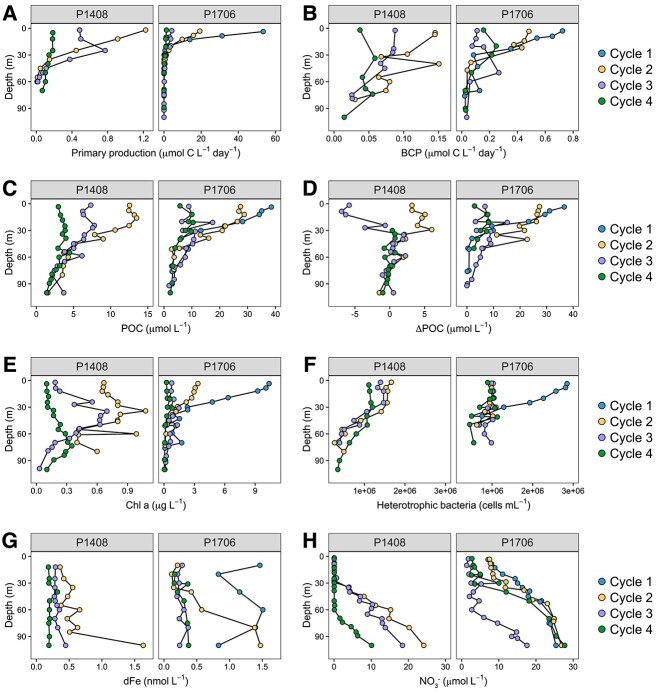
Depth profiles of biogeochemical parameters during each Cycle of the P1408 and P1706 CCE LTER process cruises. (A) Rates of primary production (μmol C L^−1^ day^−1^). (B) Rates of bacterial carbon production (BCP, μmol C L^−1^ day^−1^). (C) Concentrations of particulate organic carbon (POC, μmol L^−1^). (D) Concentrations of accumulated POC (ΔPOC, μmol L^−1^). (E) Concentrations of chlorophyll *a* (Chl *a*, μg L^−1^). (F) Heterotrophic bacteria cell abundances (cells mL^−1^). (G) Concentrations of dissolved Fe (dFe, nmol L^−1^). (H) Concentrations of nitrate (NO_3_^−^, μmol L^−1^). For all panels, sampling locations correspond to Cycles (Fig. 2) and values represent the mean value of all measurements taken across the duration of a Cycle (2–4 days) at a given depth. In most panels, different *x*-axis scales have been used for each cruise.

P1408 took place following the onset of anomalous warming in the CCS, and surface waters were 0.8–1.5°C above average [51, 52] (Fig. 2). Each Cycle of P1408 sampled a distinct water mass, and rates of primary production, POC concentrations, and chlorophyll *a* (Chl *a*) concentrations indicate that productivity sequentially decreased from Cycle 2 to Cycle 4 (Fig. 3A,C and E). Overall, measures of production in the upper 30 m were below summer mean values for this region as determined by the long-term California Cooperative Oceanic Fisheries Investigation (CalCOFI) dataset (Fig. S1A). In particular, concentrations of accumulated POC (ΔPOC) were low, remaining near or below zero during Cycles 3 and 4 (Fig. 3D), indicating POC concentrations were within the bottom 10th percentile of those measured over the course of the CalCOFI timeseries. Measures of secondary production, as determined by bacterial cell abundances and carbon production (BCP) also decreased sequentially from Cycle 2 to Cycle 4 (Fig. 3B and F), and there was a tight coupling between POC concentrations and measures of primary and secondary production (Fig. S1B).

During P1408, dFe concentrations were sub-nanomolar in the upper 100 m of the water column, and NO_3_^−^ concentrations were generally <1 μmol L^−1^ in the upper 30 m (Fig. 3G, H). In the CCS, Fe is likely to be the proximate limiting nutrient when NO_3_^−^:dFe ratios are >10 μmol:nmol, indicating incomplete utilization of available NO_3_^−^ due to low Fe availability [5, 6]. Fe limitation of the diatom community is indicated by Si:NO_3_^−^ ratios <1 mol:mol as a result of the preferential uptake of silicic acid by diatoms relative to NO_3_^−^ under Fe limitation [4]. At the DCM, NO_3_^−^:dFe ratios exceeded a value of 10 μmol:nmol only at Cycle 3 whereas Si: NO_3_^−^ ratios <1 mol:mol were observed at both Cycles 2 and 3 (Table 1).

P1706 took place after the anomalously warm period of 2014–2016 in the CCS and captured a representative upwelling event from its source at the coast as it aged and moved offshore (Fig. 2). Overall, measures of productivity were significantly higher during P1706 compared to P1408 and exceeded summer mean values for the region in the near surface (Fig. 3A-E, Fig. S1A). Measures of primary production generally decreased from Cycle 1 to Cycle 4; however, POC concentrations remained elevated in both Cycles 1 and 2 (Fig. 3C). Across all Cycles, POC concentrations were nearly equal to ΔPOC concentrations (Fig. 3D), an indicator of upwelled, higher-density water masses at the surface, fueling high levels of production. Measures of BCP followed a similar pattern to that of POC (Fig. 3B), and POC was again strongly correlated with measures of primary and secondary production (Fig. S1B).

Residual NO_3_^−^ concentrations at the surface were detected across the study region during P1706 (Fig. 3H), indicating the potential for widespread Fe limitation of the photosynthetic community. Average surface dFe concentrations at the beginning of Cycle 1 were  ~ 2 nmol L^−1^ but quickly decreased to sub-nanomolar levels moving offshore beyond the shelf break (Fig. 3G). NO_3_^−^:dFe ratios >10 μmol:nmol and Si:NO_3_^−^ ratios <1 mol:mol were observed in surface waters at Cycles 2–4 (Table 1).

### *In*  *situ* gene expression of the bacterial community

Across the dataset, an average of 53.3 ± 44.3 million high-quality read pairs were generated per sample, yielding an average of 8.8 ± 3.1 million mRNA read pairs per sample. Across the P1408 dataset, this ranged from 7.5 to 16.3 million total read pairs per sample, with 4.3–12.0 million read pairs per sample attributed to mRNA. Across the P1706 dataset, 74.2–138 million total read pairs per sample were generated. However, due to inefficient rRNA removal, 1.4–18.8 million mRNA read pairs were obtained per sample, on par with the P1408 dataset. mRNA reads were merged and co-assembled into a metatranscriptome, across which 3 096 711 unique ORFs were detected. Of these, 1 275 520 (41.2%) could be assigned a functional annotation and 99% of functionally annotated ORFs were also assigned a taxonomic annotation. Unannotated ORFs were sparingly expressed; 1 375 843 (75.5%) of the unannotated ORF set recruited <50 reads across the entire dataset. Of the annotated ORF set, 245 850 unique ORFs (19.3%) were determined to be likely bacterial proteins and used for downstream analysis. The relative abundance of total mRNA reads attributed to *Bacteria* was overall higher in P1408, but with the exception of P1408 Cycle 2, a majority of mRNA reads mapped to ORFs belonging to *Eukaryota* (Fig. S2).

**Figure 4:**
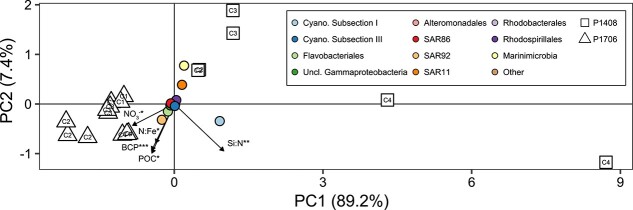
*In*  *situ* gene expression of the bacterial community during the P1408 and P1706 CCE LTER process cruises. Ordination plot displays the principal component analysis of vst-normalized mRNA read abundances belonging to a bacterial taxonomic group aggregated at the order level. The ordination of *in*  *situ* samples collected at the onset of each Fe-addition experiment are displayed as open symbols while the ordination of the most abundant bacterial orders from these samples are displayed as colored points. Replicate samples are displayed and labelled with corresponding Cycle number. Arrows display the surface linear fitted vectors of continuous environmental variables to the ordination space. Direction of arrows corresponds with the direction in the ordination space towards which a given environmental variable increases most rapidly, and the length of arrows is proportional to the *R*^2^ value of the fit between the variable and ordination space. (***) *P*  value < 0.001, (**) *P * value < 0.01, (*) *P*  value < 0.05.

Principal component (PC) analysis of *in*  *situ* bacterial mRNA expression showed a distinct separation between P1408 and P1706 along PC1, accounting for 89.2% of the total variation between samples (Fig. 4). Increases in rates of BCP, POC concentrations, NO_3_^−^ concentrations, and the NO_3_^−^:dFe ratio correlated (*P* value <0.05, linear surface fit) with the ordination of samples from P1706 (*R*^2^ = 0.53, 0.48, 0.40, and 0.34, respectively), whereas the Si:NO_3_^−^ ratio positively correlated with the ordination of samples from P1408 (*R*^2^ = 0.85). Ordination of the most abundant bacterial orders associated *Cyanobacteria*, *Marinimicrobia*, SAR11, *Rhodospirillales*, and *Rhodobacterales* with P1408 along PC1 whereas SAR92, *Flavobacteriales*, SAR86, a group of unclassified *Gammaproteobacteria*, and *Alteromonadales* were affiliated with P1706, consistent with the contrast in productivity between these two years. During P1408, cyanobacterial transcriptional activity was dominated by *Prochlorococcus* (75%–96%). Similar patterns in taxonomic distributions were observed based on the relative abundance of mRNA and 16S rRNA reads (Fig. S3). The taxonomic distribution of the phytoplankton community also reflected the differences in productivity between P1408 and P1706 (Fig. S4). *Cyanobacteria* contributed to 26.7 ± 23.8% of mRNA reads of photosynthetic taxa in P1408, whereas diatoms were prevalent during P1706 (36.6 ± 15.1%).

### Differential gene expression of the heterotrophic bacterial community in response to Fe additions

**Figure 5:**
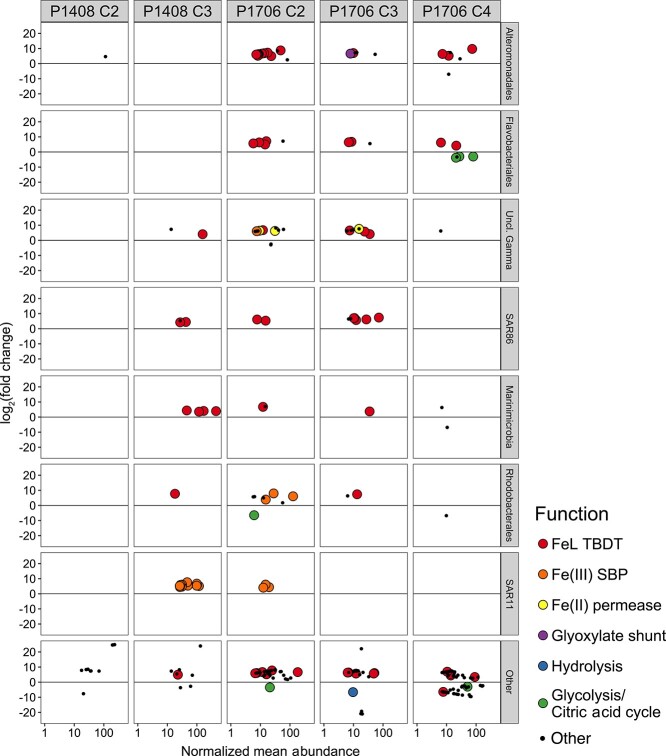
Differential gene expression of the heterotrophic bacterial community detected in response to Fe additions across the P1408 and P1706 study regions. The log_2_fold-change in expression for a given gene is plotted against its normalized mean abundance on a log_10_ scale for a given Cycle where differential gene expression was detected in response to Fe additions and separated according to the most abundant bacterial taxonomic orders. *Cyanobacteria* are included in the “other” category. Log_2_fold-changes were calculated based on ORF abundances in unamended treatments relative to those with Fe additions following 24 hours of incubation such that a positive value indicates upregulation of a given ORF under low-Fe conditions. Only ORFs with an *FDR* < 0.05 are displayed (*n* = 2 for P1408 and *n* = 3 for P1706). Colors indicate ORFs known to be Fe stress biomarkers (Fig. 1). The complete list of annotated, differentially expressed genes for each of the five Cycles displayed can be found in Dataset S1. Differential gene expression in response to Fe additions (*FDR* < 0.05) was not detected in P1408 Cycle 4 or P1706 Cycle 1. Abbreviations are consistent with those used throughout the text and KEGG/pfam orthology identifiers used for functional annotations are as follows – FeL TBDT: K02014, Fe(III) SBP: K02012, Fe(II) permease: K04759, K07243, and pfam09375, glyoxylate shunt: K01637 and K01638, hydrolysis: K03791, glycolysis/citric acid cycle: K00123, K00239, K01624, and K01682.

During each of the seven Cycles sampled (Fig. 2), experiments were conducted under *in*  *situ* temperature and light conditions to track the transcriptional response of the surface microbial community to Fe additions. In five of the seven experiments conducted, statistically significant differential gene expression by bacteria was detected in response to Fe additions, and this response was dominated by heterotrophic bacteria (Fig. 5, Dataset S1). Differential gene expression is presented as the log_2_fold-change in ORF abundance, comparing control treatments to Fe-amended treatments such that a positive fold-change indicates higher expression under unamended, low-Fe conditions. Results compare expression after the control and Fe-addition treatments were incubated for 24 hours to account for changes in gene expression due to growth or potential bottle effects.

Across all Cycles, genes from Fe acquisition pathways represented 45 ± 22% of the total number of differentially expressed genes but just 0.8 ± 0.1% of the total number of unique bacterial ORFs detected *in*  *situ*. The significant enrichment of Fe transport genes within the differentially expressed gene set (*P*  value < 0.001, hypergeometric distribution) indicates the transcriptomic response of the heterotrophic bacterial community was a result of Fe availability and suggests that the *in*  *situ* community was experiencing Fe stress. At P1408 Cycle 4 and P1706 Cycle 1, statistically significant differential gene expression was not detected in response to Fe additions by any member of the bacterial community, indicating that Fe was not a primary limiting nutrient to the heterotrophic bacterial community in these Cycles. When considering the ordination of all bacterial ORFs detected following 24 hours of incubation (including those that were not differentially expressed), the dissimilarity of the transcript pools between Cycles remained greater than the dissimilarity between experimental treatments within each Cycle (Fig. S5), indicating that Fe additions did not cause large shifts in the overall dynamics of the microbial community within 24 hours.

During P1408, differential expression by heterotrophic bacteria in response to Fe additions was strongest in Cycle 3 (Fig. 5). Differentially expressed genes (*n* = 30) consisted almost entirely of those encoding known Fe transport pathways, which were upregulated in unamended treatments. These included genes encoding the solute binding protein (SBP) of Fe(III) ATP-binding cassette transport (ABCT) systems (K02012) from members of the SAR11 clade as well as TonB-dependent transporters (TBDT) for the acquisition of FeL complexes (K02014) from an unclassified group of *Gammaproteobacteria*, SAR86, *Marinimicrobia*, and *Rhodobacterales*. A smaller number of differentially expressed genes (*n* = 11), primarily of unknown function, were also detected in Cycle 2. Despite its significant contribution to the *in*  *situ* bacterial transcript pool during P1408, differential gene expression in response to Fe additions was not detected for *Prochlorococcus* in any Cycle.

During P1706, a strong transcriptomic response by heterotrophic bacteria to Fe additions was detected across Cycle 2 (*n* = 79 genes), Cycle 3 (*n* = 45 genes), and Cycle 4 (*n* = 77 genes) (Fig. 5). Differentially expressed genes consisted of a wider diversity of both taxonomic and functional annotations compared to that of P1408 but continued to include genes encoding known Fe transport pathways and were indicative of an initially Fe-stressed community. During Cycle 2, genes encoding Fe(III) SBPs (K02012) were upregulated in unamended conditions and came from members of the SAR11 clade, *Rhodobacterales*, and unclassified *Gammaproteobacteria*. Genes encoding FeL TBDTs (K02014) were upregulated across Cycles 2–4 and came from unclassified members of *Gammaproteobacteria* as well as from the orders *Alteromonadales*, *Flavobacteriales*, SAR86, *Marinimicrobia*, *Oceanospirillales*, *Methylococcales*, and *Sphingomonadales*. A putative Fe(II) transport system (pfam09375) from unclassified members of *Gammaproteobacteria* was also upregulated in Cycles 2 and 3.

In line with culture studies, genes involved in carbon metabolism also showed patterns of differential expression consistent with Fe stress during P1706. During Cycles 2 and 4 there was a downregulation of the Fe-containing enzymes succinate dehydrogenase (K00239), aconitase (K01682), formate dehydrogenase (K00123), and Class II fructose-bisphosphate aldolase (K01624) coming from members of *Flavobacteriales*, *Rhodobacterales*, *Sphingobacteriales*, and the SAR116 clade. Additionally, genes from *Alteromonadales* encoding enzymes of the glyoxylate shunt— malate synthase (K01638) and isocitrate lyase (K01637)—were upregulated in unamended conditions during Cycles 2 and 3. Finally, the hydrolytic enzymes chitinase and β-glycosidase showed decreased expression in unamended treatments during Cycles 3 and 4.

### POC:dFe ratios across the CCS

**Figure 6:**
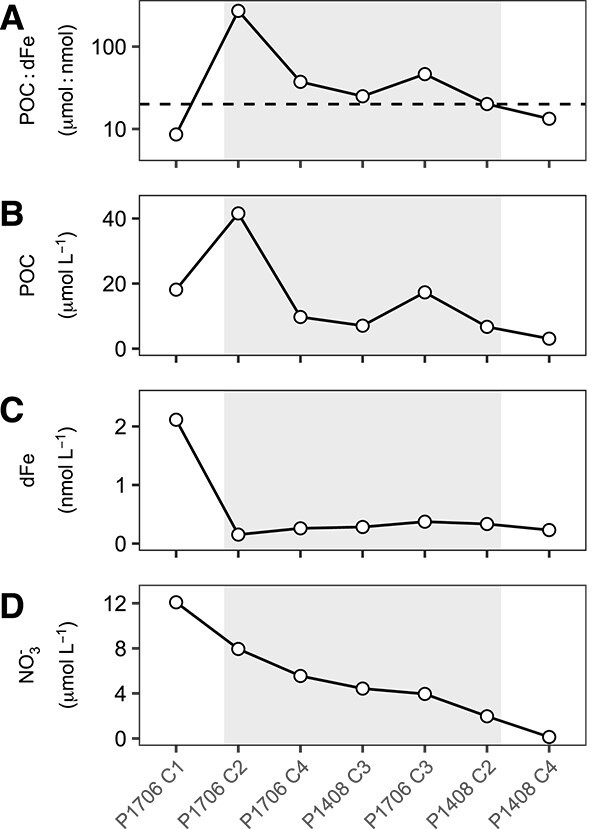
Relative availability of Fe and organic carbon as an indicator of Fe stress in heterotrophic bacterial communities of the CCS. Plots display the (A) ratios of the concentrations of particulate organic carbon to dissolved Fe (POC:dFe, μmol:nmol), (B) concentrations of particulate organic carbon (POC, μmol L^−1^), (C) concentrations of dissolved Fe (dFe, nmol L^−1^), and (D) concentrations of nitrate (NO_3_^−^, μmol L^−^^1^) at the onset of each Fe-addition experiment conducted in the CCS. Experiments are arranged in order of decreasing NO_3_^−^ concentrations as an indicator of relative upwelling strength at each Cycle. Shaded gray area indicates Cycles at which Fe stress of the heterotrophic bacterial community was indicated based on the detection of differential gene expression in response to Fe additions. The *y*-axis in (A) is on a log_10_ scale and the dashed line marks the 20 μmol:nmol POC:dFe threshold.

Across these Fe addition experiments, the ratio of POC to dFe concentrations (POC:dFe) emerged as a consistent indicator of heterotrophic Fe stress. In all Cycles where the POC:dFe ratio exceeded 20 μmol:nmol at the onset of the experiment, differential gene expression of the bacterial community was detected in response to Fe additions (Fig. 6). Sampling locations can be arranged by NO_3_^−^ concentrations, indicating the influence of coastal upwelling at each Cycle. Within this frame of reference, POC:dFe ratios peaked in the transition zone of the California Current (P1408 Cycles 2–3 and P1706 Cycles 2–4), whereas the lowest values were present at the extremes of both highly productive (P1706 Cycle 1) and oligotrophic (P1408 Cycle 4) waters. The POC:dFe ratio during Cycle 2 of P1408 lies right at the proposed Fe-stress threshold of 20 μmol:nmol, which is also reflected in the modest transcriptomic response to Fe additions detected here relative to other Cycles. The POC:dFe ratio was also examined across three GEOTRACES transects spanning the Peru-Humboldt Current System, the Atlantic sector of the Southern Ocean, and the North Atlantic (Supplemental Results). This analysis demonstrated consistent patterns of the POC:dFe ratio and identified additional regions of the global surface ocean where the POC:dFe ratio exceeds 20 μmol:nmol (Fig. S6, Table S1).

### *In*  *situ* expression of Fe stress biomarkers

**Figure 7:**
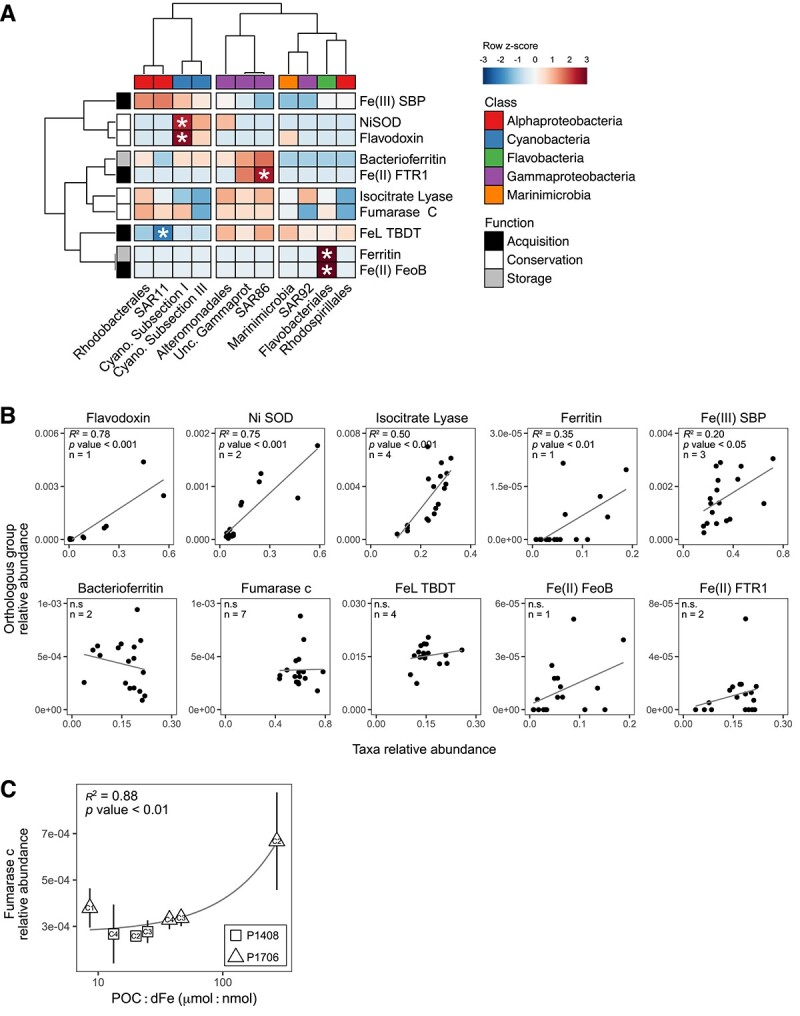
*In*  *situ* expression of bacterial Fe stress biomarkers. (A) Heatmap displays normalized ORF abundances from orthologous groups of known Fe stress biomarkers for a given bacterial order across all *in*  *situ* samples. Values are displayed as row *z*-scores and bacterial orders (*x*-axis) and orthologous groups (*y*-axis) are clustered based on Pearson correlations using Ward’s minimum variance method. Column color bar denotes bacterial class for each order while row color bar denotes whether the given orthologous group is involved in Fe acquisition, storage, or conservation. Cells marked with an asterisk are those for which the *in*  *situ* expression was 1.96σ greater than or less than the row mean value, indicating expression by a single bacterial order that falls outside of the 95% confidence interval for a given orthologous group. (B) The relative abundance of mRNA reads from specific Fe stress biomarkers within the total transcript pool is plotted against the relative abundance of all mRNA reads within the transcript pool from bacterial orders that significantly contributed to the expression of the Fe stress biomarker. *In*  *situ* expression from replicate samples at every Cycle are plotted from both P1408 and P1706. Linear regressions are displayed with the *R*^2^ and *P* values given. Linear regressions denoted with “n.s.” are not statistically significant (*P* value >0.05). The number of bacterial orders included in the taxa relative abundance for each plot is given by *n* as determined from (A) and Fig. S7. (C) The relative abundance of fumarase c within the total transcript pool is plotted against the POC:dFe ratio measured at each Cycle. Mean values from replicate samples at each Cycle are displayed and error bars represent ±1 standard deviation from the mean (*n* = 2 for P1408 and *n* = 3 for P1706). A single linear regression is displayed with the *R*^2^ and *P* values given. The *x*-axis is displayed on a log_10_ scale. For all panels, abbreviations are consistent with those used throughout the text and KEGG orthology identifiers used for functional annotations are as follows – NiSOD: K00518, isocitrate lyase: K01637, fumarase c: K01679, Fe(III) SBP: K02012, FeL TBDT: K02014, ferritin: K02217, bacterioferritin: K03594, flavodoxin: K03839, Fe(II) FeoB: K04759, Fe(II) FTR1: K07243.

Given the indication of heterotrophic bacterial Fe stress based on differential gene expression, patterns in the *in*  *situ* expression of Fe stress biomarkers (Fig. 1) were investigated. The expression of Fe stress biomarkers was detected *in*  *situ* at every Cycle (Fig. 7 and Fig. S7), and the abundance of specific orthologous groups clustered according to expression by taxonomic class (Fig. 7A, Fig. S7B and C, Supplemental Results). Hierarchical clustering (Fig. 7A) divides the expression of Fe stress biomarkers by order into two primary groups. The first contains *Alphaproteobacteria* and *Cyanobacteria* identified by the above-average expression of Fe(III) SBPs (K02012). The second consists of *Gammaproteobacteria*, *Flavobacteria*, *Marinimicrobia*, and *Rhodospirallales* characterized by the above-average expression of FeL TBDTs (K02014). In the case of nickel-containing superoxide dismutase (NiSOD) (K00518), flavodoxin (K03839), Fe(II) permeases (K04759, K07243), and ferritin (K02217), expression by a single bacterial order fell outside the 95% confidence interval of the mean expression across all taxa. Thus, the *in*  *situ* distribution of specific Fe stress biomarkers often significantly correlated with shifts in the taxonomic distribution of the overall transcript pool (*P* value <0.05, linear regression) (Fig. 7B). However, the abundance of bacterioferritin (K03594), fumarase c (K01679), FeL TBDTs, and Fe(II) permeases did not significantly correlate with the overall taxonomic distribution (Fig. 7B). Of these orthologous groups, fumarase c, an Fe-free metabolic replacement within the citric acid cycle (Fig. 1), was expressed by the highest diversity of bacterial orders as well as the most evenly across bacterial orders (Fig. S7C). It was also the only Fe stress biomarker detected *in*  *situ* that significantly correlated with the POC:dFe ratio (*P* value <0.05, linear regression) (Fig. 7C). ORFs coming from siderophore biosynthetic pathways were also searched for across this dataset (pfam04183, pfam00501, pfam00668, and pfam00550). However, only two ORFs, homologs to *dhbF* and *entF* (coming from bacillibactin and enterobactin biosynthetic pathways, respectively), were identified and detected sparingly *in*  *situ*, recruiting fewer than 10 reads each.

## Discussion

### Differential gene expression indicates the nutritional status of marine heterotrophic bacteria

In this work, we examined the differential gene expression of the heterotrophic bacterial community across the southern CCS in response to Fe additions, and the observed expression patterns indicate that this community was experiencing Fe stress under *in*  *situ* conditions. This response was particularly prevalent during P1706 where biogeochemical parameters and growth experiments also demonstrated widespread Fe limitation of the photosynthetic community [53, 54]. However, the disproportionate response of genes specific to Fe acquisition in the heterotrophic bacterial community, along with the rapid timeframe of this response, suggests that heterotrophic bacteria were specifically responding to Fe availability rather than secondary effects resulting from an Fe-limited photosynthetic community. The concurrent Fe limitation of both the heterotrophic and photosynthetic microbial communities supports the idea that these two groups are indeed competing for scarce Fe resources in the sunlit waters of the surface ocean [14, 15, 45, 55].

The diversity of Fe stress biomarkers within the genomes of heterotrophic bacteria, particularly those involved in cellular Fe transport, represents an opportunity as well as a challenge in efforts to detect *in*  *situ* Fe stress within this community. The results presented here highlight the effectiveness of short-term incubations using differential gene expression in response to Fe additions as a means to detect *in*  *situ* Fe stress. The expression of Fe stress biomarkers, particularly that of Fe transport systems, was sensitive to Fe additions. Furthermore, these additions did not result in large or artificial changes to the overall transcript pool within 24 hours–*in situ* environmental conditions and the overall dynamics of the microbial community appeared to be largely preserved. Even so, the ability to detect *in*  *situ* Fe stress using transcriptomic techniques independently of incubations would allow for a better understanding of the nutritional status of heterotrophic bacteria on a larger scale. Across this study, the expression of Fe stress biomarkers by the heterotrophic bacterial community was consistently detected *in*  *situ*. However, the distribution of specific biomarkers was often largely a factor of the taxonomic distribution of the overall transcript pool, likely due to adaptations in Fe transport capacity of specific taxa to distinct ecological niches [37, 56, 57]. For example, the expression of specific Fe acquisition pathways was distinct between background-adapted clades such as SAR11 and copiotrophic groups like *Alteromonadales* and *Flavobacteriales,* as has been observed in previous genomic and transcriptomic analyses [31, 32, 36]. Thus, as a first order, the expression of a given bacterial Fe stress biomarker *in*  *situ* may be the result of trophic state. Methods for determining an Fe stress response *in*  *situ* will, therefore, need to control for microbial community composition and the taxonomic distribution of specific Fe stress biomarkers [57, 58]. In order to evaluate Fe stress at a community-wide scale based on *in*  *situ* expression, biomarkers such as fumarase c, which was expressed by a wide diversity of bacterial taxa in this study and correlated with trends in the relative availability of carbon and Fe, may make an effective choice.

### The POC:dFe ratio as a biogeochemical proxy for Fe limitation of heterotrophic bacteria

Based on the transcriptomic response of the heterotrophic community to Fe additions across P1408 and P1706, marine heterotrophic bacterial activity appears to be a factor of organic carbon as well as Fe availability. Indeed, where Fe limitation of the heterotrophic community has been tested previously, responses to Fe additions are often only observed with the simultaneous addition of organic carbon, suggesting co-limitation between Fe and carbon is common [15, 23, 24, 27]. The range of productivity in the CCS across P1408 and P1706 created a natural laboratory in which to explore this hypothesis. Under coastal upwelling conditions, nutrients delivered to surface waters fueled high rates of primary production, increasing the availability of organic matter to heterotrophic bacteria and likely relieving carbon limitation. However, as these upwelled water masses moved offshore, Fe availability decreased relative to the availability of freshly produced organic matter, and the heterotrophic bacterial community exhibited signs of Fe stress. Consistently high levels of production in eastern boundary upwelling systems such as the CCS may mean that Fe limitation of the heterotrophic bacterial community is a persistent feature in these systems (Supplemental Results, Fig. S6). In contrast, in regions such as the Southern Ocean or oligotrophic gyres where primary production is chronically nutrient-limited, heterotrophic bacteria may predominantly experience carbon limitation, being driven to Fe limitation only during episodic bloom events [27].

In order to quantify the relationship between carbon and Fe availability as controlling factors of heterotrophic bacterial growth, we explored the ratio of POC:dFe concentrations as a biogeochemical proxy for *in situ* Fe stress that can complement molecular-based approaches. The ratio of nutrients available within a system can be a useful indicator of nutrient limitation [6, 7, 59, 60], and the threshold indicative of limitation for such a proxy will depend on the ratio at which two nutrients are utilized by dominant microbial groups within a given environment. In this dataset, POC:dFe ratios >20 μmol:nmol were indicative of Fe-limited heterotrophic bacterial communities. Values above this threshold, therefore, represent conditions where carbon is in excess relative to Fe based on cellular requirements. Thus, even within an Fe-limited phytoplankton community that may respond to Fe additions with increased productivity, the heterotrophic bacterial community would be expected to respond to Fe rather than carbon availability when the *in situ* POC:dFe ratio exceeds 20 μmol:nmol.

Relatively few studies have attempted to characterize the Fe requirements of marine heterotrophic bacteria either in culture or natural communities [8–13, 15, 61]. However, across these studies, values for the cellular C:Fe stoichiometry of heterotrophic marine bacteria range from ~1 to >2000 μmol:nmol – likely varying as a factor of growth conditions, lifestyle strategies, the potential for luxury Fe storage, and methodologies employed. However, culture studies focused on strains from the copiotrophic groups *Alteromonas* and *Pseudoalteromonas* report C:Fe stoichiometries between ~7 and 62 μmol:nmol under replete growth conditions [13, 61]. During P1706 in particular, copiotrophic strains such as these comprised a significant portion of the bacterial transcript pool and rates of BCP and growth [62, 63] were elevated, altogether indicating a fast-growing bacterial community where carbon limitation had been relieved. Under these conditions, POC:dFe ratios exceeding values of 20 μmol:nmol would be consistent with a nutrient regime indicative of Fe limitation, driven by the relatively high Fe demands (lower C:Fe ratio) of copiotrophic members of the microbial community. Although there are no current estimates available for the cellular Fe demand of the SAR11 clade, this group also responded to Fe additions at POC:dFe ratios >20 μmol:nmol, suggesting similar Fe requirements for this ubiquitous clade of heterotrophic bacteria. The reported cellular C:Fe quotas of copiotrophic bacteria are similar to those of marine diatoms [64], further highlighting the competition for Fe between the dominant microbial groups present during P1706. In contrast, the lack of a transcriptional response from *Prochlorococcus* during P1408 is consistent with the lower cellular Fe requirements reported for this cyanobacterial group [65, 66]. A better understanding of cellular quotas of Fe and carbon in specific groups of heterotrophic marine bacteria will be critical to furthering our understanding of the nutritional status of the microbial community throughout the marine environment.

Determining bioavailable pools of both organic carbon and Fe will be another important consideration in establishing an appropriate biogeochemical proxy for *in*  *situ* Fe limitation of heterotrophic bacteria. Although POC is a combination of both living microbial biomass as well as detrital organic material—a portion of which will be available for degradation and consumption by heterotrophic bacteria, POC concentrations have been shown to be a reliable indicator of freshly produced carbon within the CCS [67]. In contrast, labile dissolved organic carbon, accounting for ~50% of marine net primary production [68], is processed via the microbial loop on the timescale of minutes to days [69] —making it difficult to capture fluctuations in the availability of this pool of organic matter. Thus, although POC is not necessarily a direct measure of the carbon available to heterotrophic bacteria, we propose that its production and accumulation in the surface ocean is an accurate reflection of the amount of labile carbon within the system. Likewise, the dFe pool is a complex mixture of different forms of Fe, which are not uniformly available to microorganisms. However, changes in the concentration of dFe are thought to broadly correlate with the bioavailability of Fe within a system [70]. Furthermore, a wide range of taxa exhibited signs of Fe stress across this study, regardless of niche specialization in transport capacity for either inorganic Fe or FeL complexes. The applicability of the POC:dFe ratio as an indicator of Fe stress within the heterotrophic bacterial community across other ocean ecosystems will require further testing (Fig. S6). However, the CCS encompasses a wide range of environmental conditions and associated microbial communities, spanning multiple orders of magnitude in productivity from the edge of the oligotrophic subtropical gyre to highly productive coastal upwelling environments—making this study a promising starting point.

### Potential consequences of heterotrophic bacterial Fe limitation on carbon cycling in the marine environment

Fe is an important cofactor of carbon metabolism in heterotrophic bacteria [16]. Therefore, given that heterotrophic bacteria are major facilitators of particle degradation and organic matter remineralization in the marine environment, Fe availability to this community may be expected to have downstream effects on remineralization processes and carbon export efficiencies. This may be particularly relevant in environments like the CCS, where sinking particles are the main contributor to carbon export [71]. Although the bulk growth response to Fe additions was not measured, changes in gene expression in this study suggest Fe availability impacted central carbon metabolism and growth via mechanisms consistent with previous work. For example, the reduced expression of Fe-containing enzymes within glycolysis, the citric acid cycle, and the electron transport chain under low Fe conditions has been observed in cultured isolates where it has been associated with reduced rates of cellular respiration and growth [13, 30, 72–75]. Additionally, expression of the glyoxylate shunt is linked to Fe-limiting conditions [13, 29, 30, 76–78]. The glyoxylate shunt bypasses the two steps within the citric acid cycle where carbon is lost as CO_2_ and additional reducing agents are generated (Fig. 1). Typically, the glyoxylate shunt is associated with the metabolism of fatty acids and allows intermediates from the citric acid cycle to be diverted to biosynthesis pathways [79, 80]. Its role under Fe-limiting conditions remains intriguing. Previous culture work suggests that the glyoxylate shunt helps cells to compensate for the reduction in growth resulting from Fe limitation, perhaps by directing electron flow through succinate dehydrogenase while bypassing Complex I of the electron transport chain [29]. Complex I has the highest Fe requirement of all respiratory proteins and can potentially harbor up to 50% of the total cellular Fe quota [16].

Whether due to the reallocation of carbon from respiration to biomass production or simply an overall reduction in total carbon demand due to reduced growth, bacterial Fe limitation would be expected to impact the fate of fixed carbon within an Fe-limited ecosystem, possibly leaving a higher percentage of organic carbon available for export or transfer to higher trophic levels. Fe limitation of phytoplankton is currently linked to higher export efficiencies within the CCS [53], including during P1706 [54], and has been attributed to increased silica ballasting of diatoms under Fe limitation [81]. Reduced rates of respiration due to Fe limitation of heterotrophic bacteria offers a complementary mechanism by which export efficiencies could be increased in Fe-limited systems, which will merit further investigation.

## Conclusion

Carbon and Fe are tightly coupled in the metabolism of marine heterotrophic bacteria. A thorough understanding of the bacterial requirements of both of these nutrients will be a challenging yet important step in understanding the cycling of organic matter in the marine environment. Based on the transcriptional response of the heterotrophic bacterial community in a series of Fe-addition incubations, we suggest that this community is subject to *in*  *situ* Fe stress. We found that the potential for Fe limitation within the heterotrophic bacterial community is the greatest during periods of high productivity with elevated organic matter availability but low Fe concentrations, as indicated by the ratio between POC:dFe. We hypothesize that this is largely driven by an increase in the activity of copiotrophic taxa that respond to high levels of available organic matter, thereby increasing the Fe demand of the heterotrophic bacterial community in support of increased levels of carbon metabolism. Patterns of gene expression under Fe limitation were characterized by high expression of Fe transport systems but also by shifts in the expression of enzymes within central carbon metabolism, suggesting that Fe limitation of heterotrophic bacteria results in changes to respiration and growth. Future work will be needed in order to determine the exact relationship between carbon and Fe requirements for given heterotrophic groups and, ultimately, the effects of these dynamics on the efficiency of the marine biological carbon pump.

## Supplementary Material

Manck_etal_SI_final_040824_wrae061

Manck_etal_DatasetS1_final_040824_wrae061

## Data Availability

All sequence data are openly available under NCBI BioProject PRJNA1071118 with BioSample IDs SAMN39672528-SAMN39672581. Environmental datasets generated during the P1408 and P1706 cruises can be found at the CCE LTER Datazoo repository, https://oceaninformatics.ucsd.edu/datazoo/ and at the Environmental Data Initiative Repository, https://portal.edirepository.org/nis/browseServlet?searchValue=CCE.
