# Supplementary material for: Iron limitation of heterotrophic bacteria in the California Current System tracks relative availability of organic carbon and iron
Source: ISME J. 2024 Apr 16;18(1):wrae061. doi: 10.1093/ismejo/wrae061 (PMC11069385; doi:10.1093/ismejo/wrae061)
Supplement: Manck_etal_SI_final_040824_wrae061 [file manck_etal_si_final_040824_wrae061.pdf]

Supplementary Information

Dataset S1: Excel file with expression levels, functional, and taxonomic annotations of bacterial ORFs found to be differentially expressed in response to Fe-additions during the CCE LTER P1408 and P1706 process cruises as displayed in Figure 5 of the main manuscript.

Supplementary Materials and Methods

Chlorophyll a*, particulate organic carbon, and macronutrients*

Water column profiles for particulate organic carbon (POC) and chlorophyll *a* (Chl *a*) were collected on the upcasts of rosette deployments equipped with 12 L Niskin bottles. Water samples for Chl *a* measurements were collected in amber bottles and filtered onto 25 mm glass fiber filters (GF/F, Whatman). Filters were then extracted in 90% acetone for 24 hours at -20°C and subsequently analyzed using a Turner Designs 10-AU Fluorometer fitted with a red-sensitive photomultiplier tube. For POC measurements, 4 L of water was filtered onto 25 mm pre-combusted glass fiber filters, wrapped in pre-combusted foil, and stored in liquid nitrogen until analysis onshore. Onshore, inorganic carbon was removed by acidifying filters with HCl vapor followed by oven drying overnight, and samples were analyzed on a Costech Elemental Analyzer. Concentrations of accumulated POC (ΔPOC) were calculated according to previously published methods (1). Briefly, ΔPOC is equal to the difference between the *in situ* POC concentration and a background POC concentration set as the minimum 10th percentile value of density-binned measurements from the CalCOFI time series study area subset into four regions: inshore south, inshore north, the California Current, and offshore.

Water column samples for dissolved inorganic macronutrients were collected concurrently with samples for dissolved iron (dFe) as described below and frozen at -20°C until analysis onshore. Onshore, concentrations of soluble reactive silica (Si), dissolved nitrite + nitrate (NO_2^- + NO_3^-), and dissolved nitrite (NO_2^-) were measured with colorimetric assays on a QuAAtro continuous segmented flow autoanalyzer (SEAL Analytical) (2). Nitrate (NO_3^-) concentrations were determined by taking the difference between the NO_2^- + NO_3^- concentration and NO_2^- alone.

Dissolved Fe

Water column samples for dFe were collected using a powder-coated rosette equipped with 5 L Niskin-X bottles (Ocean Test Equipment) deployed on a coated hydrowire and subsequently processed inside a Class 100 trace metal clean van by filtering through an inline 0.2 μm Acropak 200 capsule filter (VWR International) into acid-washed (trace metal grade nitric and hydrochloric acid) low-density polyethylene bottles (Nalgene). Filtered samples were acidified to pH 1.7-1.8 using concentrated hydrochloric acid (Optima grade) and stored at room temperature until analysis onshore. Onshore, samples were processed as described previously (3–5). Briefly, dFe was oxidized to Fe(III) for 1 hour with 10 mM H_2O_2 (Optima grade), buffered in-line with ammonium acetate to pH ~3.5, and selectively pre-concentrated onto a chelating column (Toyopearl® AF-Chelate-650M). dFe was eluted from the column using 0.14 M hydrochloric acid (Optima grade) and measured by chemiluminescence using flow-injection analysis and detection by photomultiplier tube (Hamamatsu Photonics). The standardization of dFe used a matrix-matched standard curve (0, 0.4, 0.8, 3.2, 10 nmol L^{-1}) using low-Fe surface Pacific seawater (0.4 nmol L^{-1}). Standards were treated identically to samples. Accuracy was assessed by repeated measurements of GEOTRACES coastal (GSC) and open Pacific Ocean (GSP) reference

seawater samples. Our measurements of GSC gave dFe = 1.506±0.148 nmol L^{-1} (n = 23, consensus 1.535±0.115 nmol L^{-1}). Our measurements of GSP gave dFe = 0.195±0.029 nmol L^{-1} (n = 20, consensus 0.155±0.045 nmol L^{-1}). Consensus values are from the July 2019 compilation (https://www.geotraces.org/wp-content/uploads/2020/03/2019_Consensus_Values_2009_samples.pdf). Precision within each analytical session, determined by replicated analyses of an in-house large volume surface Pacific seawater (0.40 nmol L^{-1}) and GEOTRACES reference seawater, was typically ±5% or better. For the duration of these analyses, the average limit of detection (defined as 3x the standard deviation of the blank) was 0.036 nmol L^{-1} (n = 43).

POC:dFe ratios

POC:dFe ratios (μmol:nmol) were calculated for the P1408 and P1706 datasets using *in situ* POC and dFe measurements collected at the onset of each incubation experiment as described above. POC and dFe concentrations from the GEOTRACES GP16, GIPY04, and GA03 cruises were downloaded from the GEOTRACES Intermediate Data Product 2021 (6). Details on sample collection and analysis can be found within respective publications describing these data (7–11). POC concentrations used in this work represent the sum of both the small (1-51 μm) and large (> 51μm) size fractions described in previous work. For the results presented here, values from discrete measurements along each transect were interpolated using Data-Interpolating Variational Analysis (DIVA) in Ocean Data View (12). Values from the resulting interpolated grids were then used to calculate the POC:dFe ratios displayed in Figure S6.

Primary production

During P1408, rates of net primary production were determined with ^{14}C *in situ* incubations. During each day of a given Cycle, water was collected at six depths spanning the euphotic zone and transferred to triplicate 250 mL polycarbonate bottles and an additional 250 mL dark bottle. Bottles were spiked with $H^{14}CO_3^-$ and incubated for 24 h on the *in situ* drifter array at the respective sampling depths, beginning and terminating at ~04:00 local time. Samples were filtered onto glass fiber filters, acidified for 24 h, and subsequently placed in liquid scintillation cocktail for counting on a liquid scintillation counter to determine ^{14}C incorporation into POC. During P1706, samples for determining rates of net primary production based on ^{14}C incorporation were not available. Primary production was therefore estimated from measurements of ambient light, nutrients, and chl *a* as described previously (13).

Bacterial carbon production

Water column samples for bacterial carbon production (BCP) measurements were collected on the upcasts of rosette deployments equipped with 12 L Niskin bottles. Measurements were conducted with technical quadruplicates (P1408) or triplicates (P1706) with duplicate (P1408) or singlet (P1706) TCA-killed controls. For each technical replicate, 1.7 mL of seawater was incubated with 20 nM ^{3}H-leucine for 1 hour at approximately 10°C. Following incubation, all samples were killed with 5% TCA and processed using the centrifugation method described previously (14). Samples were then frozen at -20°C and analyzed onshore with a Beckman LS6000A liquid scintillation counter. Rates of leucine incorporation were converted to rates of carbon production assuming 3.1 kg C mol^{-1} leucine (15).

Bacterial cell abundances

Water column samples for bacterial cell abundances were collected on the upcasts of rosette deployments equipped with 12 L Niskin bottles. 2 mL aliquots of whole seawater were preserved with 0.5% final concentration of paraformaldehyde and stored in liquid nitrogen until analysis on shore. Samples were analyzed at the Flow Cytometry Facility at the University of Hawai‘i at Mānoa School of Ocean and Earth Science and Technology. Samples were thawed and stained with Hoechst 33342 and analyzed with a Beckman-Coulter EPICS Altra flow cytometer with dual lasers. All samples were spiked with fluorescent beads to normalize fluorescence and scattering properties. Raw data were processed with the FlowJo software (Treestar Inc.), and estimates of heterotrophic bacteria, *Prochlorococcus*, and *Synechococcus* were determined by differences in light scatter and fluorescence emission.

Pigment analysis

Samples for pigment concentrations were collected from the near surface (2-5 m) on the upcasts of rosette deployments equipped with 12 L Niskin bottles. Between 500 mL – 2 L of whole seater (depending on *in situ* fluorescence values measured during the corresponding CTD cast) were filtered onto 25 mm glass fiber filters under low vacuum pressure. Filters were then stored in liquid nitrogen until analysis on shore. On shore, filters were extracted in the dark and on ice in acetone for a total of one hour, grinding the filter after the initial 30 minutes of extraction. Extracts were then analyzed for pigment concentrations by reverse-phase high pressure liquid chromatography (HPLC) on an Agilent 1200 Series Gradient HPLC system using a C8 reverse-phase silica column. Pigments were separated using a flow rate of 1 mL min^{-1} and a 12 minute gradient from 0% to 40% B followed by a 24 minute gradient to 100% B which was then maintained for an additional 4 minutes. Solvent A consisted of methanol:acetonitrile:acidic

pyridine in a 50:25:25 volumetric ratio, and solvent B consisted of methanol:acetonitrile:acetone in a 20:60:20 volumetric ratio. Chlorophyll contributions by the major phytoplankton groups present were calculated according to the primary and ancillary ratio approach (16) using the concentrations of class-specific accessory pigments as determined above. This was implemented in the CHEMTAX v1.95 software using pigment ratios from the northern Baja Californian Peninsula (17) as the input matrix.

16S and 18S rRNA amplicon sequencing

The 16S V4V5 hypervariable region and the 18S V9 hypervariable region of rRNA was amplified via PCR from cDNA using the TruFi DNA polymerase (Azura Genomics) and the 515F/926R primer pair in the case of 16S rRNA amplification and the 1389F/1510R primer pair in the case of 18S rRNA amplification. During P1408, the cDNA template used for PCR amplification was the same as that used for the preparation of the metatranscriptomic libraries, generated from the RNA extractions of the *in situ* samples described in the main text. During P1706, cDNA template for PCR amplification was generated in the same manner but came from *in situ* surface samples collected ~18 hours following the collection of those used for the *in situ* metatranscriptomic libraries described in the main text. PCR amplicons were visualized by gel electrophoresis in a 1.5% agarose gel and purified with Agencourt AMPure XP beads. PCR products were pooled and sequenced on the MiSeq platform (Ilumina) at the Institute for Genomic Medicine at the University of California, San Diego. Quality controlled reads were analyzed with the DADA2 (18) pipeline within Qiime2 (19) to generate a count table of exact amplicon sequence variants (ASVs) detected in each sample. ASVs were assigned taxonomy

using the native implementation of the naïve Bayesian classifier method within DADA2 using the Silva (16S rRNA) and pr2 (18S rRNA) databases.

Supplementary Results and Discussion

Expression of Fe transport systems in the marine environment is characterized by taxonomic niche specialization

The *in situ* expression of genes from specific orthologous groups of Fe stress biomarkers across P1408 and P1706 clustered according to taxonomic class (Main Text Figure 7, Figure S7). For example, the expression of solute binding proteins (SBPs) for the transport of Fe(III) through ATP-binding cassette transporters (ABCTs) (K02012) was dominated by *Alphaproteobacteria* during both P1408 and P1706 (Main Text Figure 7, Figure S7). During P1408, this consisted largely of expression by SAR11 accounting for 54.7 ±7.3% of the Fe(III) SBP pool. During P1706, ORFs from *Rhodobacterales* contributed significantly to the expression of Fe(III) SBPs across all Cycles (48.9 ±1.0%) in addition to SAR11 (31.6 ±11.0%). Across both cruises, ORFs encoding Fe(III) SBPs from *Rhodobacterales* consisted almost entirely of those from the pelagic roseobacter *Rhodobacterales* sp. HTCC2255. ORFs from *Cyanobacteria* could also be detected in the Fe(III) SBP pool during P1408 (9.0 ±5.4%). In contrast, the expression of TonB-dependent transporters (TBDTs) for the acquisition of larger FeL complexes (K02014) was dominated by members of *Gammaproteobacteria* and *Marinimicrobia*. ORFs from SAR86 and *Alteromonadales* were detected across both cruises in high relative abundance of the total FeL TBDT pool (36.0 ±17.4% and 25.2 ±12.3%, respectively). ORFs encoding FeL TBDTs from *Marinimicrobia* were prevalent during P1408 (39.0 ±21.0%), consistent with the overall increase in the relative abundance of ORFs from *Marinimicrobia* during this cruise (Figure S3). Outside

of these groups, contributions to the FeL TBDT pool could also be detected from *Flavobacteriales* and *Rhodospirillales* during P1706. While the expression of TBDTs for the acquisition of Fe-ligand complexes, which includes Fe-siderophore complexes, was prevalent, the expression of siderophore biosynthesis genes was not widely detected in either P1408 or P1706. Siderophores are organic ligands which bind strongly to Fe and can be produced by some marine bacteria as a means to scavenge Fe from the environment. In culture, siderophore biosynthetic pathways are found to be highly expressed in low-Fe conditions. The absence of ORFs from siderophore biosynthetic pathways in this dataset may be a result of their low overall abundance across marine metagenomes and reference databases (20,21), potentially resulting in expression levels that were not captured by the depth of sequencing achieved in this work. Finally, across these samples, ORFs enabling the acquisition of Fe(II) via the FeoB permease (K04759) came primarily from members of *Flavobacteriales* (89.2 ±20.4%) and were detected almost exclusively during P1706 (Figure S7). However, the capacity for Fe(II) transport via the FTR1-like permease (K07243) was also detected in a number of samples from both cruises, and these genes were expressed exclusively by SAR86 and an unclassified group of *Gammaproteobacteria*.

In addition to Fe transport systems, ORFs related to Fe storage and conservation also showed distinct patterns in the taxonomic distribution of their expression (Main Text Figure 7, Figure S7). The capacity for Fe storage via the expression of bacterioferritn (K03594) or ferritin (K02217) came largely from SAR86, an unclassified group of *Gammaproteobacteria* and *Flavobacteriales* and to a lesser extent from *Alteromonadales*, *Rhodobacterales*, and *Cyanobacteria*. *Gammaproteobacteria* dominated the expression of isocitrate lyase (K01637)

across both cruises but with contributions from *Rhodobacterales* also detected in P1706. Isocitrate lyase is a non-Fe containing enzyme found within the glyoxylate shunt (Main Text Figure 1), an alternative pathway for carbon metabolism which has been observed to be highly expressed by marine bacteria in low-Fe conditions (22–25). Fumarase c (K01679), another Fe-free metabolic replacement found within the citric acid cycle (Main Text Figure 1) was expressed by a wider diversity of bacteria including *Cyanobacteria*, *Flavobacteriales*, unclassified *Gammaproteobacteria*, *Alteromonadales*, SAR86, SAR11, and *Rhodobacterales*. Finally, a nickel-containing superoxide dismutase (NiSOD, K00518) (Main Text Figure 1) and flavodoxin (K03839), an Fe-free metabolic replacement for ferredoxin in the redox reactions of photosynthesis, were expressed primarily by *Cyanobacteria*, but members of *Alteromonadales* also contributed to NiSOD expression during P1706.

As a result of the taxonomic separation in the expression of specific orthologous groups of Fe stress biomarkers, the overall expression of these orthologous groups differed between P1408 and P1706 in accordance with the shifts in the dominant taxonomic groups contributing to the mRNA transcript pool (Main Text Figure 7b, Figure S3a, Figure S7a). Notably, ORFs encoding the FeoB permease for Fe(II) transport (K04759) along with ORFs for isocitrate lyase (K01637), fumarase c (K01679), and ferritin (K02217) were detected in higher abundances across P1706. The expression of FeL TBDTs (K02014) along with the FTR1-like permease (K07243), a non-specific divalent metal transporter capable of Fe(II) acquisition (26), also clustered with orthologous groups highly expressed during P1706. In contrast, genes encoding the SBP of the Fe(III) ABCT system (K02012) were highly expressed during P1408 compared to P1706 along with NiSOD (K00518), flavodoxin (K03839), and bacterioferritin (K03594).

Fe is present in the marine environment in a wide array of chemical forms – including both inorganic and organically-complexed species spanning soluble to particulate size ranges (27,28). Thus, Fe availability is a factor of both concentration as well as the ability of microorganisms to access specific forms of Fe through dedicated cellular transport pathways (Main Text Figure 1). This means that the availability of specific Fe substrates in a given environment has the potential to impart control on genomic content, resulting in niche specialization in transport capacity across taxonomic groups of marine heterotrophic bacteria. Indeed, a growing body of research suggests that transport capacity of organic matter as well as trace metals by marine bacteria is a result of niche specialization with important implications for ecological dynamics and biogeochemical cycling (25,29–35).

In the current study, the observation of *in situ* gene expression across a wide spectrum of environmental conditions allowed us to investigate how this idea of niche specialization in trace metal acquisition may play out in natural communities. We observed that while the dominant pathways for Fe acquisition expressed by the heterotrophic bacterial community can change across time and space (Figure S7a), the major taxonomic groups expressing a given pathway remained consistent (Main Text Figure 7, Figure S7) and was primarily divided by taxon association with either copiotrophic or oligotrophic environments. This lends strong evidence that niche specialization in trace metal acquisition, as predicted by comparative genomics (20,30,36), plays out at the ecosystem scale and offers important insight into the bioavailable forms of Fe present in a given environment. The expression of TBDTs by copiotrophic groups during P1706 supports the prevalence of organically-complexed Fe, acquired via TBDTs, in

environments enriched in organic matter. In contrast, inorganic Fe(III), acquired via ABCTs expressed primarily by background-adapted clades during P1408, is likely to be the favorable Fe source in oligotrophic conditions. Of particular note from this study was the active expression of permeases for inorganic Fe(II) acquisition during P1706 (Figure S7). The FeoB permease for Fe(II) acquisition has been found to be enriched in the genomes of *Flavobacteria* (20), and the prevalence of this copiotrophic group in the high productivity waters encountered in P1706 accounts for the distribution of this transport system. Although Fe(II) is generally scarce in the oxygenated marine environment due to an oxidation half-life on the order of minutes to days (37–39), it has been hypothesized that copiotrophic, particle-associated bacteria may encounter low-oxygen microenvironments during active periods of particle remineralization (40), potentially increasing localized concentrations of Fe(II) in these reducing environments. Direct evidence for the development of such anoxic microenvironments has been described based on cadmium sulfide precipitation (41) and models suggest that anoxic microenvironments contribute substantially to global marine anoxic conditions (42). The active expression of genes encoding Fe(II) transport systems further suggests that these reducing microenvironments can develop within particles, and, furthermore, that Fe(II) is an important source of Fe in these productive environments (43) – even when Fe(II) may not be detected by conventional means in an overall well-oxygenated system.

The ratio of POC:dFe as a biogeochemical proxy for Fe limitation of heterotrophic bacteria across other ocean basins

In order to explore the potential for Fe stress of heterotrophic bacteria across ocean ecosystems outside of the CCS, POC:dFe ratios were calculated across three GEOTRACES transects where

concurrent measurements of POC and dFe were made (Figure S6). The GEOTRACES GP16 transect traversed the Peru-Humboldt Current System, another of the four major eastern boundary coastal upwelling ecosystems (44). Similar to our observations in the CCS, in this system, POC:dFe ratios peaked away from the continental shelf as dFe concentrations rapidly decreased yet concentrations of POC remained relatively elevated (7,8). Again, both nearshore, high productivity waters and offshore, lower productivity waters exhibited the lowest POC:dFe ratios. During the GEOTRACES GIPY04 transect in late austral summer across the Atlantic sector of the Southern Ocean, POC:dFe ratios increased south of the Subtropical Front as surface concentrations of POC and dFe began to exhibit opposing trends (9,10). Along this transect, the extent of values exceeding the proposed threshold of 20 μmol:nmol throughout the upper 100 m increased to the south, reflecting the increasing depth of the surface mixed-layer. As a result, at the southern Antarctic Circumpolar Current Front, where the mixed-layer depth reached 120 m, the POC:dFe ratio throughout the entire upper 100 m exceeded 20 μmol:nmol. In contrast, across the North Atlantic subtropical gyre (NASG) as captured by the GEOTRACES GA03 transect during late autumn, POC:dFe ratios never exceeded a value of 20 μmol:nmol in the surface ocean. This is likely a result of elevated surface dFe concentrations coming from atmospheric dust deposition (45). Maximum POC:dFe values approaching the threshold value of 20 μmol:nmol were found near the DCM where dFe concentrations reached minimum values, likely as a result of biological uptake or particle scavenging (45).

The applicability of the POC:dFe ratio as an indicator of Fe stress within the heterotrophic bacterial community across other ocean ecosystems will require further testing. However, the range of environmental conditions and associated microbial communities captured in the CCS in

the current study spans a wide breadth of the variability in biogeochemical conditions found across the global surface ocean – making this study a promising starting point. Examining this ratio across the Peru-Humboldt Current System, another of the four eastern boundary upwelling systems where Fe limitation of primary production is known to develop (46), revealed similar patterns in the distribution of this ratio to that in the CCS (Main Text Figure 6). Additionally, the region where POC:dFe values exceeded 20 μmol:nmol also coincides with the highest surface concentrations of siderophores detected along this transect (47), both occurring in the transition from coastal upwelling to more oligotrophic conditions. As discussed above, siderophore biosynthetic pathways are highly expressed by marine bacteria under low-Fe conditions, and their production seems to be stimulated in carbon-enriched environments (48–50). The high abundance of siderophores detected alongside high POC:dFe ratios is consistent with the idea that the heterotrophic community was experiencing Fe stress in this transition zone and indicates that Fe stress of the heterotrophic bacterial community may be a persistent feature within eastern boundary coastal upwelling systems.

We also evaluated the POC:dFe ratio as a proxy for Fe limitation of heterotrophic bacteria across transects of the Southern Ocean and NASG (Figure S6). From the perspective of primary production, the Southern Ocean encompasses the largest Fe-limited biome in the marine environment (28). Thus, understanding the effects of Fe availability on the heterotrophic bacterial community will be important to understanding biogeochemical dynamics in this region. Our analysis of POC:dFe ratios across the Atlantic sector of the Southern Ocean revealed a large portion of the upper water column with ratios exceeding 20 μmol:nmol. Like the CCS, concentrations of bulk DOC in the upper water column of the Southern Ocean are not a reliable

indicator of the availability of organic carbon due to the constant dilution of labile DOC produced in the surface with low concentrations of DOC transported from depth via upwelling (51). Thus, POC concentrations may again be a useful indicator of labile organic carbon available in this environment. Indeed, in previous incubations near the Kerguelen Islands, heterotrophic bacteria exhibited a growth response to Fe additions exclusively in productive waters (52). In this study, measures of primary and secondary production were reflected in POC rather than DOC concentrations (53), and the response of heterotrophic bacteria to Fe additions can be divided along a POC:dFe threshold of 20 μmol:nmol (Table S1).

In contrast to HNLC environments, the direct response of heterotrophic bacteria to Fe additions has yet to be tested in the NASG. However, useful comparisons can be drawn with the North Pacific subtropical gyre (NPSG) where bacterial carbon production has been stimulated in response to Fe additions at the DCM (54) when POC:dFe ratios exceeded 20 μmol:nmol (Table S1). In both the NASG and NPSG, atmospheric dust deposition often results in higher dFe concentrations in the surface mixed-layer, and minimum values are typically observed deeper in the euphotic zone (55,56). While POC:dFe ratios never exceeded the threshold of 20 μmol:nmol during the GA03 transect in late autumn, maximum values were observed at the DCM (Figure S6), corresponding with this region of low Fe availability. In the subtropical gyres, intense microbial competition for Fe in the lower euphotic zone (57,58) may push the heterotrophic bacterial community to Fe stress at these deeper depths, particularly during productive seasons when both microbial competition for Fe and carbon availability to the heterotrophic community would be expected to increase.

Supplementary Figures and Tables

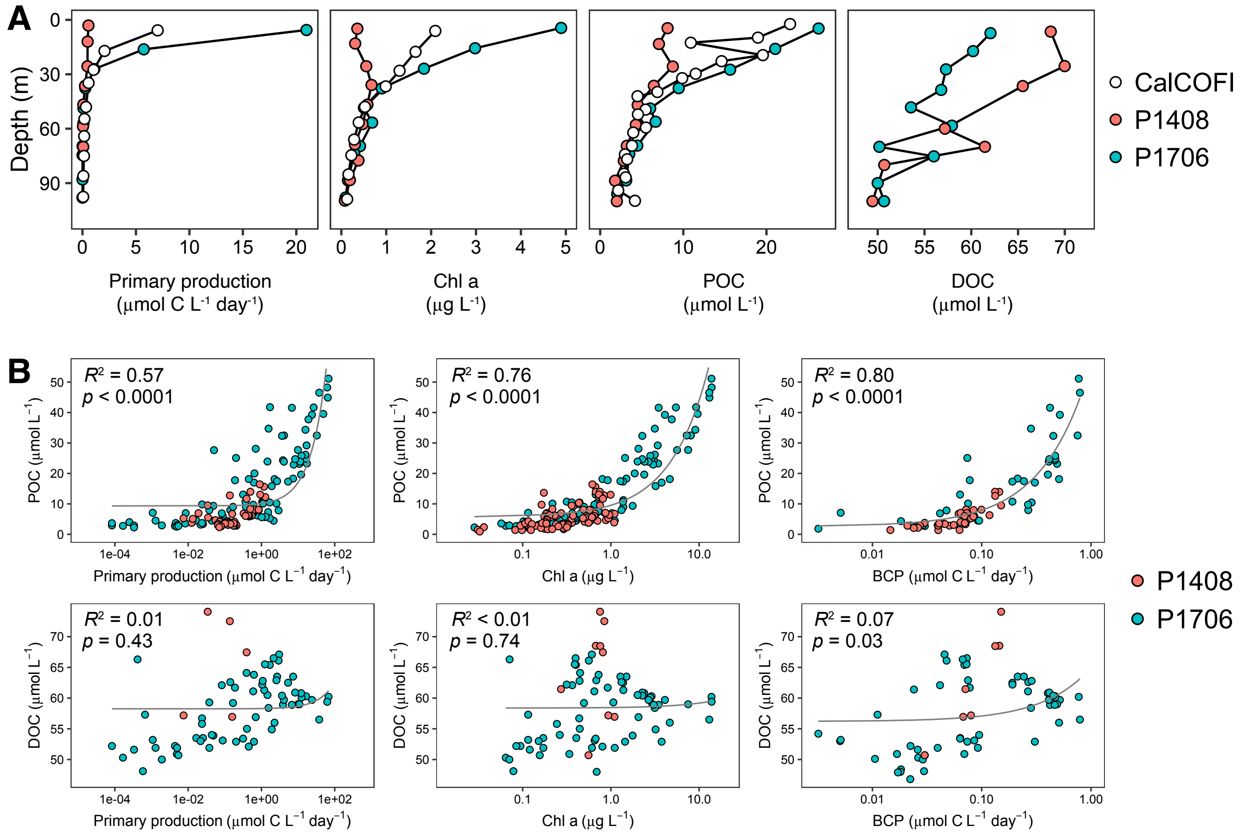

Figure S1: Measures of productivity across the P1408 and P1706 CCE LTER process cruises. **(A)** Depth profiles display rates of primary production (µmol C L^{-1} day^{-1}), concentrations of chlorophyll *a* (Chl *a*, µg L^{-1}), concentrations of particulate organic carbon (POC, µmol L^{-1}), and concentrations of dissolved organic carbon (DOC, µmol L^{-1}) during the P1408 and P1706 process cruises compared to summer mean values from the CalCOFI timeseries. Values plotted for P1408 and P1706 are mean values of those within 10 m depth bins collected across the entire cruise. Values plotted for CalCOFI are mean values of those within 10 m depth bins from the northern Lines 076.7 and 080.0 during June, July, and August across the entire timeseries (primary production: 1984-2019, Chl *a*: 1978-2020, POC: 2005-2020). DOC is not routinely measured by the CalCOFI program. **(B)** Concentrations of POC (µmol L^{-1}, top row) and DOC (µmol L^{-1}, bottom row) from the P1408 and P1706 process cruises are plotted against measures of primary and seconday production. Data from all Cycles are displayed together and colored according to cruise. Lines display linear regressions with the R^2 and p values of the regressions also displayed. A single regression for all data across both cruises has been calculated. The *x*-axes are on a $\log_{10}$ scale. For (A) and (B) DOC concentrations are only available from Cycle 2 during P1408.

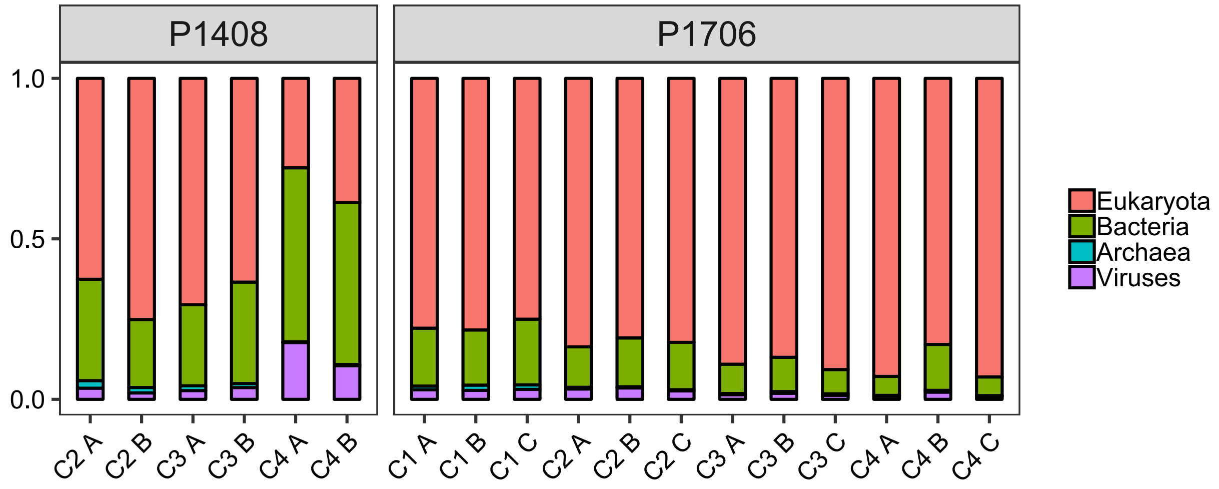

Figure S2: The taxonomic distribution of all annotated ORFs detected *in situ* across the P1408 and P1706 CCE LTER process cruises is displayed based on the relative abundance of mRNA reads assigned to each domain in a given sample.

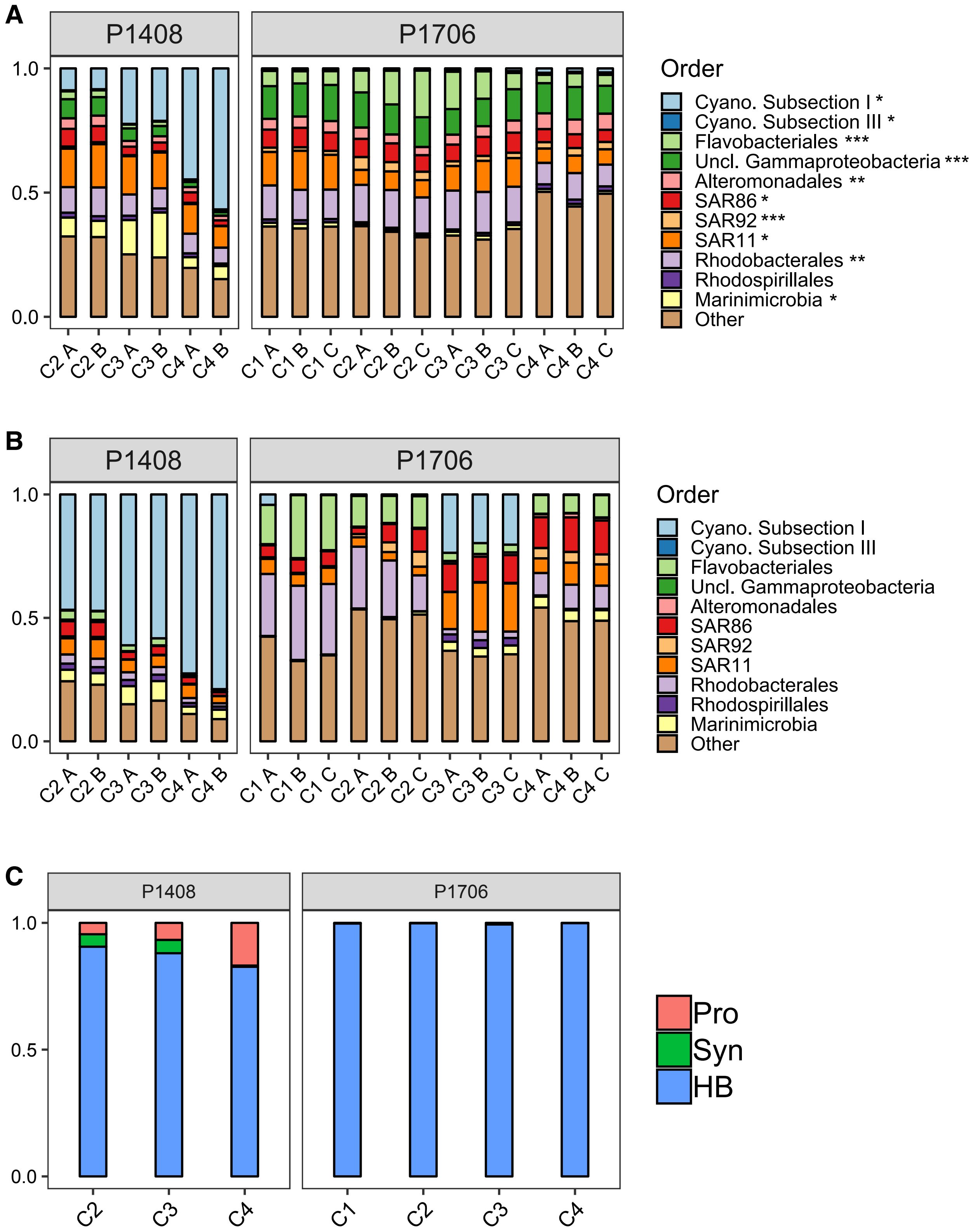

A
P1408
P1706
1.0
0.5
0.0
C2 A
C2 B
C3 A
C3 B
C4 A
C4 B
C1 A
C1 B
C1 C
C2 A
C2 B
C2 C
C3 A
C3 B
C3 C
C4 A
C4 B
C4 C
Order
Cyano. Subsection I *
Cyano. Subsection III *
Flavobacteriales ***
Uncl. Gammaproteobacteria ***
Alteromonadales **
SAR86 *
SAR92 ***
SAR11 *
Rhodobacterales **
Rhodospirillales
Marinimicrobia *
Other
B
P1408
P1706
1.0
0.5
0.0
C2 A
C2 B
C3 A
C3 B
C4 A
C4 B
C1 A
C1 B
C1 C
C2 A
C2 B
C2 C
C3 A
C3 B
C3 C
C4 A
C4 B
C4 C
Order
Cyano. Subsection I
Cyano. Subsection III
Flavobacteriales
Uncl. Gammaproteobacteria
Alteromonadales
SAR86
SAR92
SAR11
Rhodobacterales
Rhodospirillales
Marinimicrobia
Other
C
P1408
P1706
1.0
0.5
0.0
C2
C3
C4
C1
C2
C3
C4
Pro
Syn
HB

Figure S3: The taxonomic distribution of the *in situ* bacterial community across the P1408 and P1706 CCE LTER processes cruises is displayed according to **(A)** the relative abundance of all bacterial mRNA reads according to order, **(B)** the relative abundance of 16S rRNA reads according to bacterial order following amplification of the V4V5 hypervariable region from cDNA, and **(C)** the relative abundance of bacterial cell counts attributed to heterotrophic bacteria (HB), *Prochlorococcus* (Pro) or *Synechococcus* (Syn) as determined by flow cytometry. In (A) asterisks denote orders whose mean relative abundances were significantly different between P1408 and P1706 (independent Student's *t*-test, (***) $p < 0.001$, (**) $p < 0.01$, (*) $p < 0.05$).

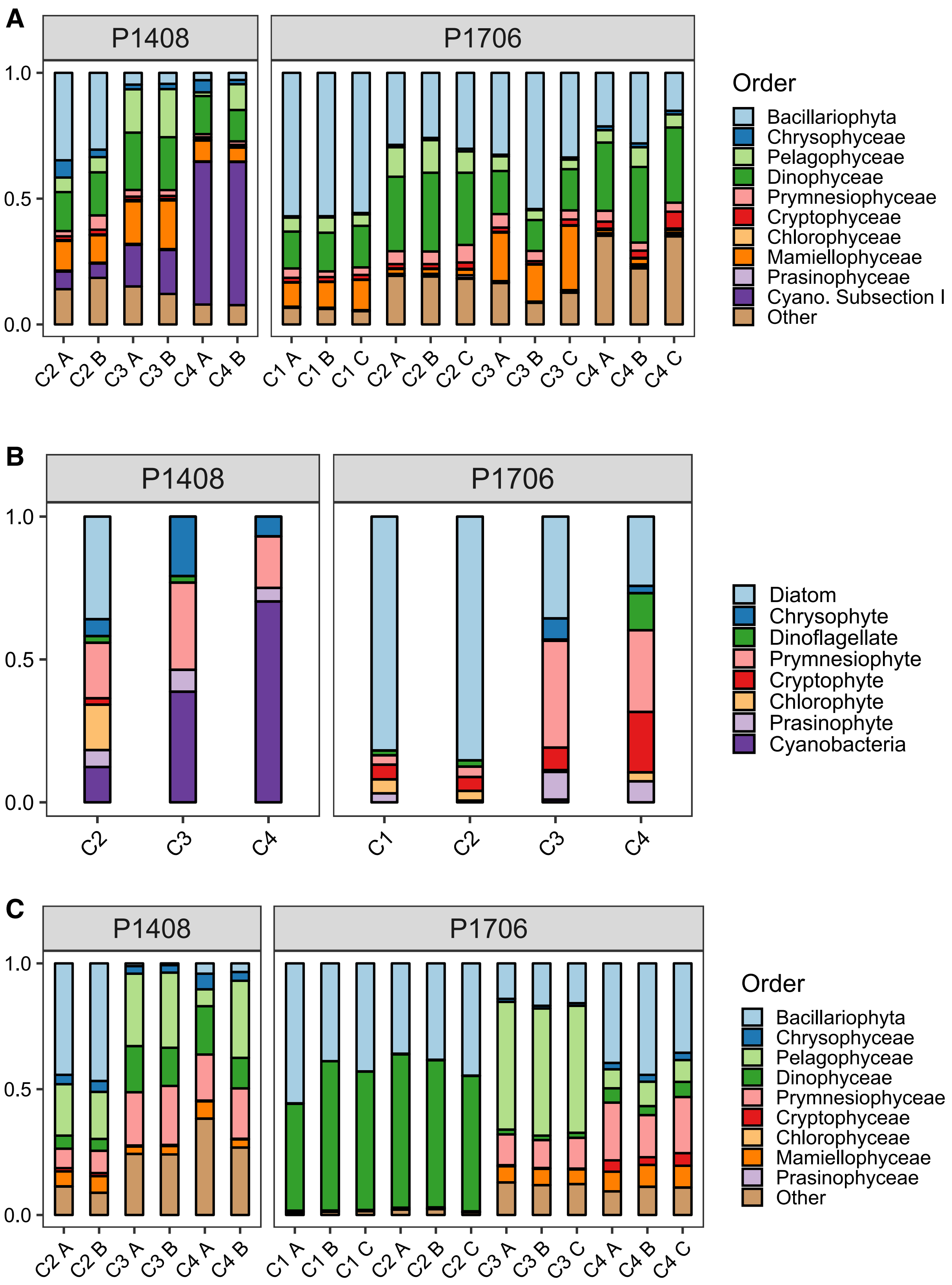
A
P1408
P1706
1.0
0.5
0.0
C2 A
C2 B
C3 A
C3 B
C4 A
C4 B
C1 A
C1 B
C1 C
C2 A
C2 B
C2 C
C3 A
C3 B
C3 C
C4 A
C4 B
C4 C
Order
Bacillariophyta
Chrysophyceae
Pelagophyceae
Dinophyceae
Prymnesiophyceae
Cryptophyceae
Chlorophyceae
Mamiellophyceae
Prasinophyceae
Cyano. Subsection I
Other
B
P1408
P1706
1.0
0.5
0.0
C2
C3
C4
C1
C2
C3
C4
Diatom
Chrysophyte
Dinoflagellate
Prymnesiophyte
Cryptophyte
Chlorophyte
Prasinophyte
Cyanobacteria
C
P1408
P1706
1.0
0.5
0.0
C2 A
C2 B
C3 A
C3 B
C4 A
C4 B
C1 A
C1 B
C1 C
C2 A
C2 B
C2 C
C3 A
C3 B
C3 C
C4 A
C4 B
C4 C
Order
Bacillariophyta
Chrysophyceae
Pelagophyceae
Dinophyceae
Prymnesiophyceae
Cryptophyceae
Chlorophyceae
Mamiellophyceae
Prasinophyceae
Other

Figure S4: The taxonomic distribution of the *in situ* phytoplankton community across the P1408 and P1706 CCE LTER process cruises is displayed based on **(A)** the relative abundance of mRNA reads from photosynthetic classes of the microbial community according to order, **(B)** the relative abundance of total chlorophyll *a* attributed to specific phytoplankton taxa based on the ratios of class-specific pigment concentrations, and **(C)** the relative abundance of 18S rRNA reads from eukaryotic photosynthetic classes according to order following amplification of the V9 hypervariable region from cDNA (excludes *Cyanobacteria*).

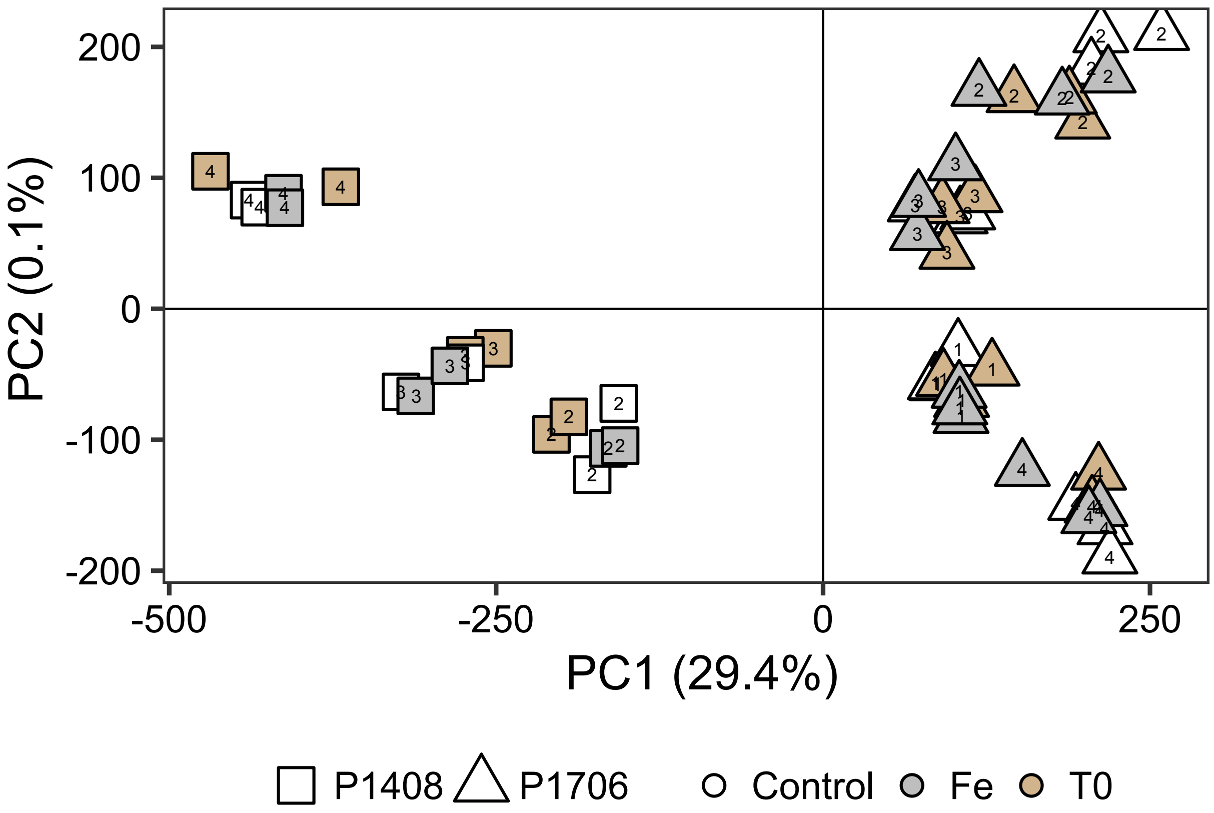

Figure S5: Expression of all bacterial ORFs detected at T0 compared to both the control and Fe-addition treatments following 24 hours of incubation under *in situ* light and temperature conditions at each Cycle for both P1408 and P1706. Ordination plot displays the principal component analysis of vst-normalized mRNA read abundances from each incubation. Squares display ordination of samples from P1408 while triangles display ordination of samples from P1706. Symbols are colored according to treatment (T0, control, Fe-addition) and labeled with Cycle number. Replicate samples are displayed.

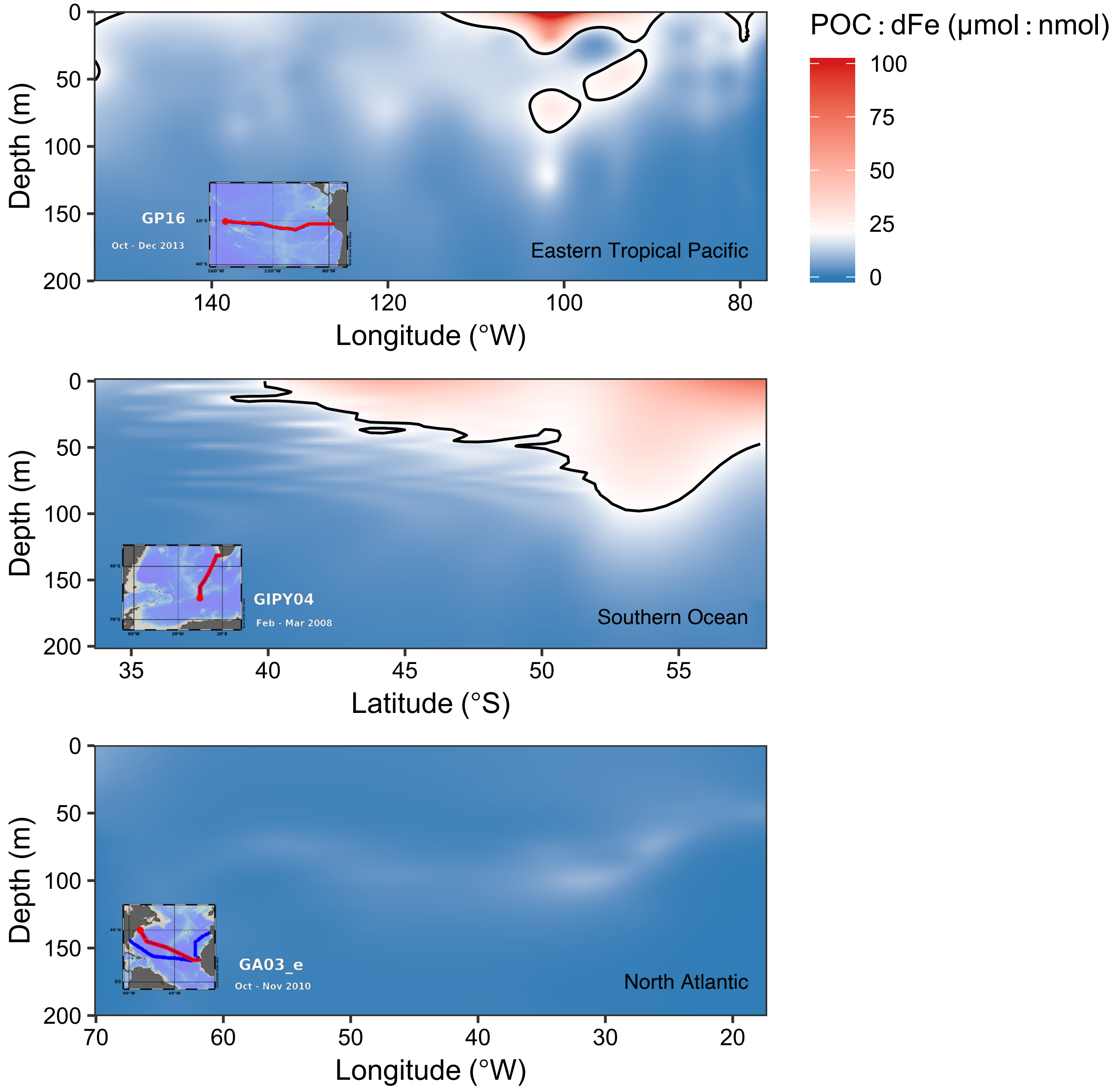

Figure S6: Section plots display POC:dFe ratios calculated in the upper 200 m of the water column across three GEOTRACES transects where POC and dFe concentrations were concurrently measured (GP16, GIPY04, and GA03). Inset figures display cruise tracks. Black contour lines mark the threshold of 20 μmol:nmol as an indicator of Fe limitation in the heterotrophic bacterial community.

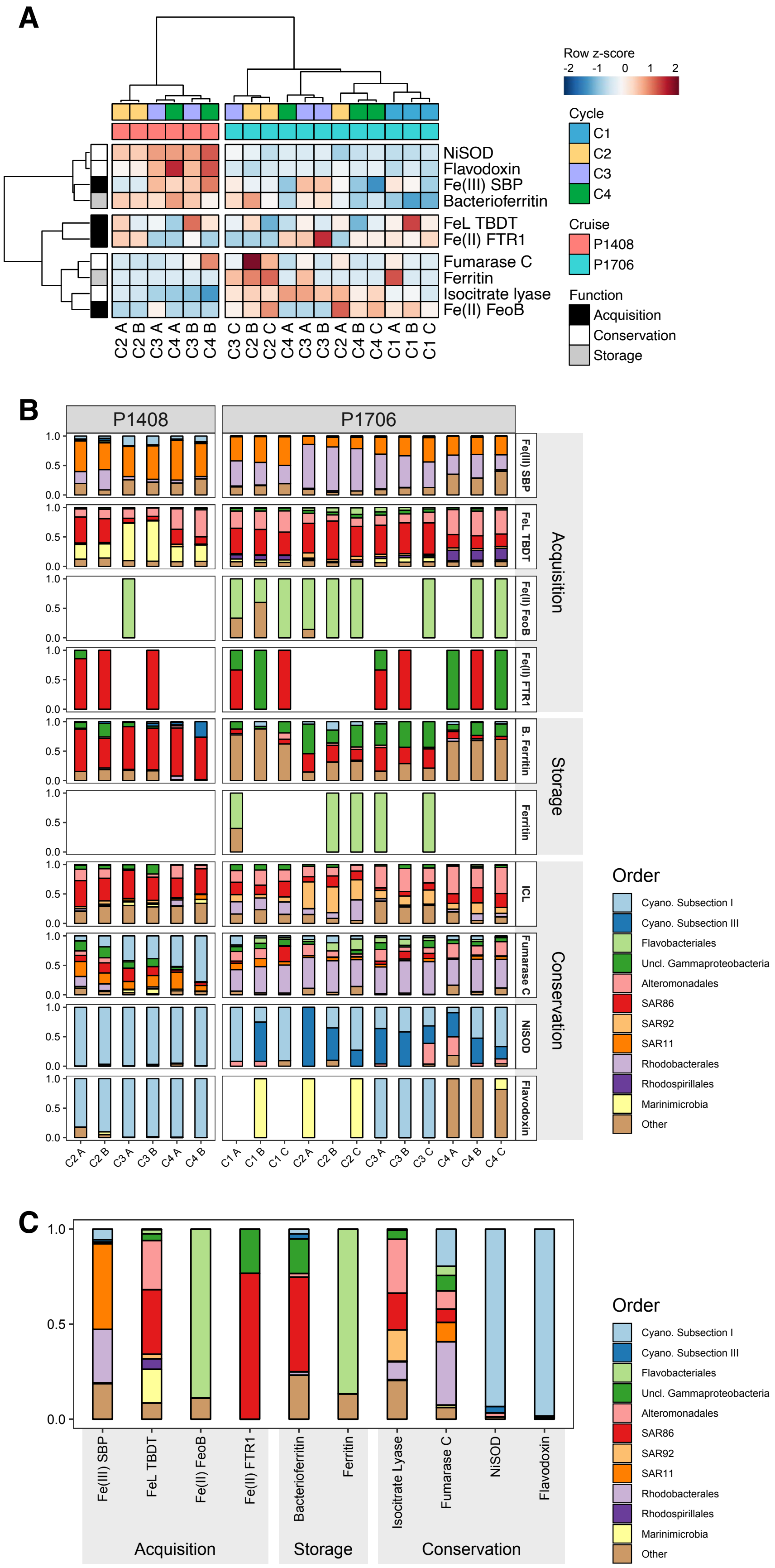
A
Row z-score
-2 -1 0 1 2
Cycle
C1
C2
C3
C4
Cruise
P1408
P1706
Function
Acquisition
Conservation
Storage
NiSOD
Flavodoxin
Fe(III) SBP
Bacterioferritin
FeL TBDT
Fe(II) FTR1
Fumarase C
Ferritin
Isocitrate lyase
Fe(II) FeoB
C2 A C2 B C3 A C4 A C3 B C4 B C3 C C2 B C2 C C4 A C3 A C3 B C2 A C4 B C4 C C1 A C1 B C1 C
B
P1408
P1706
Fe(III) SBP
FeL TBDT
Fe(II) FeoB
Fe(II) FTR1
B. Ferritin
Ferritin
ICL
Fumarase C
NiSOD
Flavodoxin
Acquisition
Storage
Conservation
1.0 0.5 0.0
C2 A C2 B C3 A C3 B C4 A C4 B
C1 A C1 B C1 C C2 A C2 B C2 C C3 A C3 B C3 C C4 A C4 B C4 C
Order
Cyano. Subsection I
Cyano. Subsection III
Flavobacteriales
Uncl. Gammaproteobacteria
Alteromonadales
SAR86
SAR92
SAR11
Rhodobacterales
Rhodospirillales
Marinimicrobia
Other
C
1.0 0.5 0.0
Fe(III) SBP
FeL TBDT
Fe(II) FeoB
Fe(II) FTR1
Bacterioferritin
Ferritin
Isocitrate Lyase
Fumarase C
NiSOD
Flavodoxin
Acquisition
Storage
Conservation
Order
Cyano. Subsection I
Cyano. Subsection III
Flavobacteriales
Uncl. Gammaproteobacteria
Alteromonadales
SAR86
SAR92
SAR11
Rhodobacterales
Rhodospirillales
Marinimicrobia
Other

Figure S7: *In situ* gene expression of bacterial ORFs known to be Fe stress biomarkers that were detected across the P1408 and P1706 CCE LTER process cruises. **(A)** Heatmap displays the vst-normalized ORF abundances of known Fe stress biomarkers across *in situ* samples collected at the onset of each Fe-addition experiment. Values are displayed as row z-scores and samples (x-axis) and orthologous groups (y-axis) are clustered based on Pearson correlations using Ward's minimum variance method. Replicate samples are displayed. Column color bars denote the cruise and Cycle number for a given sample while row color bars denote whether the given orthologous group is involved in Fe acquisition, storage, or conservation. **(B)** Bar plots display the relative abundance of mRNA reads in each *in situ* sample attributed to a given bacterial order for a given orthologous group of Fe stress biomarker. Replicate samples displayed and labeled according to Cycle number for a given cruise. **(C)** Bar plots display the relative abundance of mRNA reads across all *in situ* samples collected during both P1408 and P1706 attributed to a given bacterial order for a given Fe stress biomarker orthologous group. In (B) and (C) orthologous groups are organized according to involvement in Fe acquisition, storage, or conservation. For all panels, abbreviations are consistent with those used throughout the text and KEGG orthology identifiers used for functional annotations are as follows – NiSOD: K00518, ICL: K01637, Fumarase c: K01679, Fe(III) SBP: K02012, FeL TBDT: K02014, Ferritin: K02217, Bacterioferritin: K03594, Flavodoxin: K03839, Fe(II) FeoB: K04759, Fe(II) FTR1: K07243.

Table S1: POC:dFe ratios (µmol:nmol) from previous Fe-addition studies in the Southern Ocean and North Pacific Subtropical Gyre (NPSG).

Study	Region	Depth (m)	dFe[a] (nmol L^{-1})	POC[b] (µmol L^{-1})	POC:dFe (µmol:nmol)	Growth Response[c]
KEOPS2	Southern Ocean	40	0.13	2.37	18.2	-
KEOPS2	Southern Ocean	40	0.17	12.58	74.0	+
KEOPS2	Southern Ocean	37	0.35	7.58	21.7	+
KEOPS2	Southern Ocean	25	0.06	7.59	126.5	+
MESO-SCOPE	NPSG	104	0.06	2.31	36.7	+
MESO-SCOPE	NPSG	118	0.14	1.80	13.2	-

[a]Data from Obernosterer et al. (52) for the KEOPS2 study and Hawco et al. (54) for the MESO-SCOPE study.

[b]Data from Trull et al. (53) for the KEOPS2 study (bulk POC measurements) and Barone et al. (59) for the MESO-SCOPE study.

[c]Response of the bacterial community to Fe additions in each of the studies referenced. A plus symbol (+) denotes that there was a statistically significant increase in bacterial carbon production and/or heterotrophic bacterial cell abundances in response to Fe additions compared to unamended control treatments. A dash (-) denotes that there was not a statistically significant difference in the growth of bacteria following Fe additions compared to unamended controls. Growth response results from Obernosterer et al. (52) for the KEOPS2 study and Hawco et al. (54) for the MESO-SCOPE study.

References for Supplementary Information

1. Stephens BM, Porrachia M, Dovel S, Roadman M, Goericke R, Aluwihare LI. Nonsinking organic matter production in the California Current. Global Biogeochem Cycles. 2018;32(9):1386–405.
2. Gordon LI, Jennings JC, Ross, Andrew A J, Krest JM. A suggested protocol for continuous flow automated analysis of seawater nutrients in the WOCE hydrographic program and the Joint Global Ocean Fluxes Study. Grp Tech Rpt OSU Coll Oceanogr Descr Chem Oc. 1992;92(1):29–39.
3. King AL, Barbeau KA. Evidence for phytoplankton iron limitation in the southern California Current System. Mar Ecol Prog Ser. 2007;342:91–103.
4. King AL, Barbeau KA. Dissolved iron and macronutrient distributions in the southern California Current System. J Geophys Res. 2011;116(C3):C03018.
5. Lohan MC, Aguilar-Islas AM, Bruland KW. Direct determination of iron in acidified (pH 1.7) seawater samples by flow injection analysis with catalytic spectrophotometric detection: Application and intercomparison. Limnol Oceanogr Methods. 2006;4(6):164–71.
6. GEOTRACES Intermediate Data Product 2021 (IDP2021). NERC EDS Bristish Oceanographic Data Center NOC; 2021.
7. John SG, Helgoe J, Townsend E, Weber T, DeVries T, Tagliabue A, et al. Biogeochemical cycling of Fe and Fe stable isotopes in the Eastern Tropical South Pacific. Mar Chem. 2018;201:66–76.
8. Lam PJ, Lee JM, Heller MI, Mehic S, Xiang Y, Bates NR. Size-fractionated distributions of suspended particle concentration and major phase composition from the U.S.

GEOTRACES Eastern Pacific Zonal Transect (GP16). Mar Chem. 2018;201:90–107.

9. Abadie C, Lacan F, Radic A, Pradoux C, Poitrasson F. Iron isotopes reveal distinct dissolved iron sources and pathways in the intermediate versus deep Southern Ocean. Proc Natl Acad Sci U S A. 2017;114(5):858–63.

10. Planchon F, Cavagna AJ, Cardinal D, André L, Dehairs F. Late summer particulate organic carbon export and twilight zone remineralisation in the Atlantic sector of the Southern Ocean. Biogeosciences. 2013;10(2):803–20.

11. Lam PJ, Ohnemus DC, Auro ME. Size-fractionated major particle composition and concentrations from the US GEOTRACES North Atlantic Zonal Transect. Deep Res Part II Top Stud Oceanogr. 2015;116:303–20.

12. Schlitzer R. Ocean Data View. 2021.

13. Stukel MR, Goericke R, Landry MR. Predicting primary production in the southern California Current Ecosystem from chlorophyll, nutrient concentrations, and irradiance. bioRxiv. 2019;590240. https://doi.org/10.1101/590240.

14. Smith DC, Azam F. A simple, economical method for measuring bacterial protein synthesis rates in seawater using 3H-leucine 1. Mar Microb Food Webs. 1992;6(2):107–14.

15. Simon M, Azam F. Protein content and protein synthesis rates of planktonic marine bacteria. Mar Ecol Prog Ser. 1989;51:201–13.

16. Goericke R, Montoya JP. Estimating the contribution of microalgal taxa to chlorophyll a in the field—variations of pigment ratios under nutrient- and light-limited growth. Mar Ecol Prog Ser. 1998 Jan 15;169:97–112.

17. González-Silvera A, Santamaría-del-Ángel E, Camacho-Ibar V, López-Calderón J,

Santander-Cruz J, Mercado-Santana A. The effect of cold and warm anomalies on phytoplankton pigment composition in waters off the Northern Baja California Peninsula (México): 2007–2016. J Mar Sci Eng. 2020;8:533.

18. Callahan BJ, McMurdie PJ, Rosen MJ, Han AW, Johnson AJA, Holmes SP. DADA2: High-resolution sample inference from Illumina amplicon data. Nat Methods. 2016;13(7):581–3.

19. Bolyen E, Rideout JR, Dillon MR, Bokulich NA, Abnet CC, Al-Ghalith GA, et al. Reproducible, interactive, scalable and extensible microbiome data science using QIIME 2. Nat Biotechnol. 2019;37(8):852–7.

20. Hopkinson BM, Barbeau KA. Iron transporters in marine prokaryotic genomes and metagenomes. Environ Microbiol. 2012;14(1):114–28.

21. Manck LE, Park J, Tully BJ, Poire AM, Bundy RM, Dupont CL, et al. Petrobactin, a siderophore produced by *Alteromonas*, mediates community iron acquisition in the global ocean. ISME J. 2022;16(2):358–69.

22. Fourquez M, Devez A, Schaumann A, Guéneuguès A, Jouenne T, Obernosterer I, et al. Effects of iron limitation on growth and carbon metabolism in oceanic and coastal heterotrophic bacteria. Limnol Oceanogr. 2014;59(2):349–60.

23. Beier S, Gálvez MJ, Molina V, Sarthou G, Quéroué F, Blain S, et al. The transcriptional regulation of the glyoxylate cycle in SAR11 in response to iron fertilization in the Southern Ocean. Environ Microbiol Rep. 2015;7(3):427–34.

24. Koedooder C, Guéneuguès A, Van Geersdaële R, Vergé V, Bouget F-Y, Labreuche Y, et al. The role of the glyoxylate shunt in the acclimation to iron limitation in marine heterotrophic bacteria. Front Mar Sci. 2018;5:435.

25. Manck LE, Espinoza JL, Dupont CL, Barbeau KA. Transcriptomic study of substrate-specific transport mechanisms for iron and carbon in the marine copiotroph *Alteromonas macleodii*. mSystems. 2020;5(2):e00070-20.

26. Große C, Scherer J, Koch D, Otto M, Taudte N, Grass G. A new ferrous iron-uptake transporter, EfeU (YcdN), from *Escherichia coli*. Mol Microbiol. 2006;62(1):120–31.

27. Gledhill M, Buck KN. The organic complexation of iron in the marine environment: A review. Front Microbiol. 2012;3:69.

28. Tagliabue A, Bowie A, Boyd P, Buck KN, Johnson KS, Saito M. The integral role of iron in ocean biogeochemistry. Nature. 2017;543:51–9.

29. Reintjes G, Arnosti C, Fuchs B, Amann R. Selfish, sharing and scavenging bacteria in the Atlantic Ocean: a biogeographical study of bacterial substrate utilisation. ISME J. 2019;13(5):1119–32.

30. Hogle SL, Cameron Thrash J, Dupont CL, Barbeau KA. Trace metal acquisition by marine heterotrophic bacterioplankton with contrasting trophic strategies. Appl Environ Microbiol. 2016;82(5):1613–24.

31. Teeling H, Fuchs BM, Becher D, Klockow C, Gardebrecht A, Bennke CM, et al. Substrate-controlled succession of marine bacterioplankton populations induced by a phytoplankton bloom. Science. 2012;336(6081):608–11.

32. Buchan A, LeCleir GR, Gulvik CA, González JM. Master recyclers: features and functions of bacteria associated with phytoplankton blooms. Nat Rev Microbiol. 2014;12(10):686–98.

33. Cottrell MT, Kirchman DL. Natural assemblages of marine proteobacteria and members of the *Cytophaga-Flavobacter* cluster consuming low- and high-molecular-weight

dissolved organic matter. Appl Environ Microbiol. 2000;66(4):1692–7.

34. Datta MS, Sliwerska E, Gore J, Polz MF, Cordero OX. Microbial interactions lead to rapid micro-scale successions on model marine particles. Nat Commun. 2016;7:11965.

35. Norris N, Levine NM, Fernandez VI, Stocker R. Mechanistic model of nutrient uptake explains dichotomy between marine oligotrophic and copiotrophic bacteria. PLoS Comput Biol. 2021;17(5):e1009023.

36. Zhang R, Debeljak P, Blain S, Obernosterer I. Seasonal shifts in Fe-acquisition strategies in Southern Ocean microbial communities revealed by metagenomics and autonomous sampling. Environ Microbiol. 2023;25(10):1816–29.

37. King DW, Lounsbury HA, Millero FJ. Rates and mechanism of Fe(II) oxidation at nanomolar total iron concentrations. Environ Sci Technol. 1995;29(3):818–24.

38. Ussher SJ, Worsfold PJ, Achterberg EP, Laës A, Blain S, Laan P, et al. Distribution and redox speciation of dissolved iron on the European continental margin. Limnol Oceanogr. 2007;52(6):2530–9.

39. Hansard SP, Landing WM, Measures CI, Voelker BM. Dissolved iron(II) in the Pacific Ocean: Measurements from the PO2 and P16N CLIVAR/CO2 repeat hydrography expeditions. Deep Res Part I Oceanogr Res Pap. 2009;56(7):1117–29.

40. Alldredge AL, Cohen Y. Can microscale chemical patches persist in the sea? Microelectrode study of marine snow, fecal pellets. Science. 1987;235(4789):689–91.

41. Janssen DJ, Conway TM, John SG, Christian JR, Kramer DI, Pedersen TF, et al. Undocumented water column sink for cadmium in open ocean oxygen-deficient zones. Proc Natl Acad Sci U S A. 2014;111(19):6888–93.

42. Bianchi D, Weber TS, Kiko R, Deutsch C. Global niche of marine anaerobic metabolisms

expanded by particle microenvironments. Nat Geosci 2018 114. 2018;11(4):263–8.

43. Toulza E, Tagliabue A, Blain S, Piganeau G. Analysis of the global ocean sampling (GOS) project for trends in iron uptake by surface ocean microbes. PLoS One. 2012;7(2):e30931.

44. Chavez FP, Bertrand A, Guevara-Carrasco R, Soler P, Csirke J. The northern Humboldt Current System: Brief history, present status and a view towards the future. Prog Oceanogr. 2008;79(2–4):95–105.

45. Hatta M, Measures CI, Wu J, Roshan S, Fitzsimmons JN, Sedwick P, et al. An overview of dissolved Fe and Mn distributions during the 2010-2011 U.S. GEOTRACES north Atlantic cruises: GEOTRACES GA03. Deep Res Part II Top Stud Oceanogr. 2015;116:117–29.

46. Hutchins DA, Hare CE, Weaver RS, Zhang Y, Firme GF, DiTullio GR, et al. Phytoplankton iron limitation in the Humboldt Current and Peru Upwelling. Limnol Oceanogr. 2002;47(4):997–1011.

47. Boiteau RM, Mende DR, Hawco NJ, McIlvin MR, Fitzsimmons JN, Saito MA, et al. Siderophore-based microbial adaptations to iron scarcity across the eastern Pacific Ocean. Proc Natl Acad Sci U S A. 2016;113(50):14237–42.

48. Haygood MG, Holt PD, Butler A. Aerobactin production by a planktonic marine *Vibrio* sp. Limnol Oceanogr. 1993;38(5):1091–7.

49. Mawji E, Gledhill M, Milton JA, Zubkov M V., Thompson A, Wolff GA, et al. Production of siderophore type chelates in Atlantic Ocean waters enriched with different carbon and nitrogen sources. Mar Chem. 2011;124(1–4):90–9.

50. Bundy RM, Boiteau RM, McLean C, Turk-Kubo KA, McIlvin MR, Saito MA, et al.

Distinct siderophores contribute to iron cycling in the mesopelagic at Station ALOHA. Front Mar Sci. 2018;5:61.

51. Hansell DA. Recalcitrant dissolved organic carbon fractions. Ann Rev Mar Sci. 2013;5:421–45.

52. Obernosterer I, Fourquez M, Blain S. Fe and C co-limitation of heterotrophic bacteria in the naturally fertilized region off the Kerguelen Islands. Biogeosciences. 2015;12(6):1983–92.

53. Trull TW, Davies DM, Dehairs F, Cavagna AJ, Lasbleiz M, Laurenceau-Cornec EC, et al. Chemometric perspectives on plankton community responses to natural iron fertilisation over and downstream of the Kerguelen Plateau in the Southern Ocean. Biogeosciences. 2015;12(4):1029–56.

54. Hawco NJ, Barone B, Church MJ, Babcock-Adams L, Repeta DJ, Wear EK, et al. Iron depletion in the deep chlorophyll maximum: Mesoscale eddies as natural iron fertilization experiments. Global Biogeochem Cycles. 2021;35:e2021GB007112.

55. Fitzsimmons JN, Hayes CT, Al-Subiai SN, Zhang R, Morton PL, Weisend RE, et al. Daily to decadal variability of size-fractionated iron and iron-binding ligands at the Hawaii Ocean Time-series Station ALOHA. Geochim Cosmochim Acta. 2015;171:303–24.

56. Conway TM, John SG. Quantification of dissolved iron sources to the North Atlantic Ocean. Nature. 2014;511(7508):212–5.

57. Hogle SL, Hackl T, Bundy RM, Park J, Satinsky B, Hiltunen T, et al. Siderophores as an iron source for picocyanobacteria in deep chlorophyll maximum layers of the oligotrophic ocean. ISME J. 2022;16:1636–46.

58. Hawco NJ, Yang SC, Pinedo-González P, Black EE, Kenyon J, Ferrón S, et al. Recycling

of dissolved iron in the North Pacific Subtropical Gyre. Limnol Oceanogr. 2022;67(11):2448–65.

59. Barone B, Church MJ, Dugenne M, Hawco NJ, Jahn O, White AE, et al. Biogeochemical dynamics in adjacent mesoscale eddies of opposite polarity. Global Biogeochem Cycles. 2022;36:e2021GB007115.
